# Supplementary material for: Visible light-driven efficient palladium catalyst turnover in oxidative transformations within confined frameworks
Source: Nat Commun. 2022 Feb 17;13:928. doi: 10.1038/s41467-022-28474-7 (PMC8854557; doi:10.1038/s41467-022-28474-7)
Supplement: Supplementary file 1 — Supplementary Information [file 41467_2022_28474_MOESM1_ESM.pdf]

Supplementary Information

Visible Light-Driven Efficient Palladium Catalyst Turnover in Oxidative Transformations within Confined Frameworks

Jiawei Li,[1,2] Liuqing He,[2] Qiong, Liu,[3] Yanwei Ren,[*,1] and Huanfeng Jiang[*,1]

[1]*Key Laboratory of Functional Molecular Engineering of Guangdong Province, School of Chemistry and Chemical Engineering, South China University of Technology, Guangzhou, 510641, People's Republic of China.*

[2]*College of Chemistry and Chemical Engineering, Central South University, Changsha, 410083, People's Republic of China.*

[3]*Institute of Analysis, Guangdong Academy of Sciences (China National Analytical Center, Guangzhou), Guangzhou, 510070, People's Republic of China.*

Corresponding Author:

Y. Ren (renyw@scut.edu.cn) and H. Jiang (jianghf@scut.edu.cn)

Supplementary Methods

All of the reagents were commercially available and were used without further purification. Powder X-ray diffraction (PXRD) patterns were collected on a Bruker D8 powder diffractometer at 40kV, 40mA with Cu Kα radiation (λ=1.5406 Å), with a step size of 0.01995^o (2θ). Thermogravimetric analyses (TGA) were performed on a Q600 SDT instrument under a flow of air at a heating rate of 5 ^{o}C/min from 25-900 ^{o}C. ^{1}H NMR and ^{13}C NMR were done on a Bruker Model AM-400 (400 MHz) spectrometer. The UV-Vis diffuse reflectance spectra were collected from 200-800 nm using PE lambda 750. Infrared (IR) spectra were measured from a KBr pellets on a Nicolet Model Nexus 470 FT-IR spectrometer. The content of metal ions was determined by the inductively coupled plasma mass spectrometry (ICP-MS) (Agilent 720ES). The N_2 adsorption measurements were performed on a MicroActive ASAP 2460 systems under 77 K. The X-ray photoelectron spectroscopy (XPS) experiments were conducted using Thermo fisher Scientific with an Al-K-Alpha+ radiation source. Scanning electron microscopy (SEM) were recorded on MERLIN Compact. Transmission electron microscopy (TEM) and elemental mapping images were performed on FEI Tecnai G2 F20. The fs-TA experiments were performed on a Helios pump-probe system (Ultrafast Systems LLC) coupled with an amplified femtosecond laser system (Coherent, 35 fs, 1 kHz, 800 nm). A small portion (around 10 μJ) of the fundamental 800-nm laser pulses was focused into a 1-mm CaF_2 to generate the probe pulses (from 400 to 650 nm). The 400-nm pump pulses were generated from an optical parametric amplifier (TOPAS-800-fs). The steady-state PL measurements were carried out by a fluorescence spectrophotometer with an excitation wavelength of 400 nm. The electron paramagnetic-resonance (EPR) spectroscopy was recorded on a BRUKER ELEXSYS-II E500 CW-EPR electron paramagnetic resonance spectrometer under dark or blue LED irradiation.

Supplementary Discussion

Synthesis of [Ir(ppy)$_2$(dcbpy)]Cl (H$_2$L).

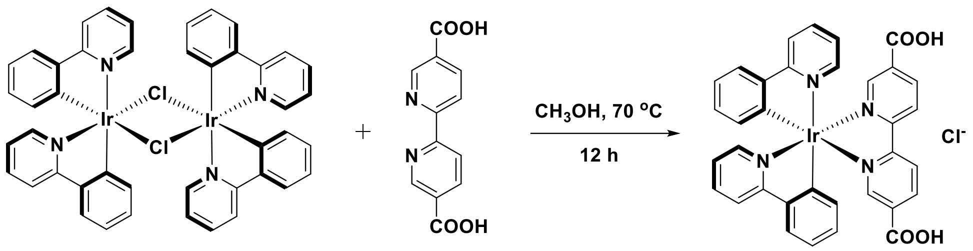

A mixture of bpy (0.2 mmol), $Ir_2(ppy)_4Cl_2$(0.12 mmol) and Na_2CO_3 (0.5 mmol) was dissolved in CH_3OH (10 mL) and then heated to reflux under stirring for 12 h. After completion, the mixture was cooled to room temperature and the solvent was removed under reduced pressure. Then, deionized water was added to dissolve the precipitation, and the filtrate was further acidified (pH = 2) with 1M HCl. The resulting precipitate was filtrated and dried to give the products (yield: 89% based on bpy). ^{1}H NMR (400 MHz, DMSO-d_6): δ 8.97 (d, J = 12 Hz, 2H), 8.57 (d, J = 12 Hz, 2H), 8.37 (s, 2H), 8.27 (d, J = 8 Hz, 2H), 7.95 (t, J = 8 Hz, 2H), 7.69 (d, J = 4 Hz, 2H), 7.16 (t, J = 8 Hz, 2H), 7.04 (t, J = 8 Hz, 2H), 6.93 (t, J = 8 Hz, 2H), 6.19 (d, J = 8 Hz, 2H).

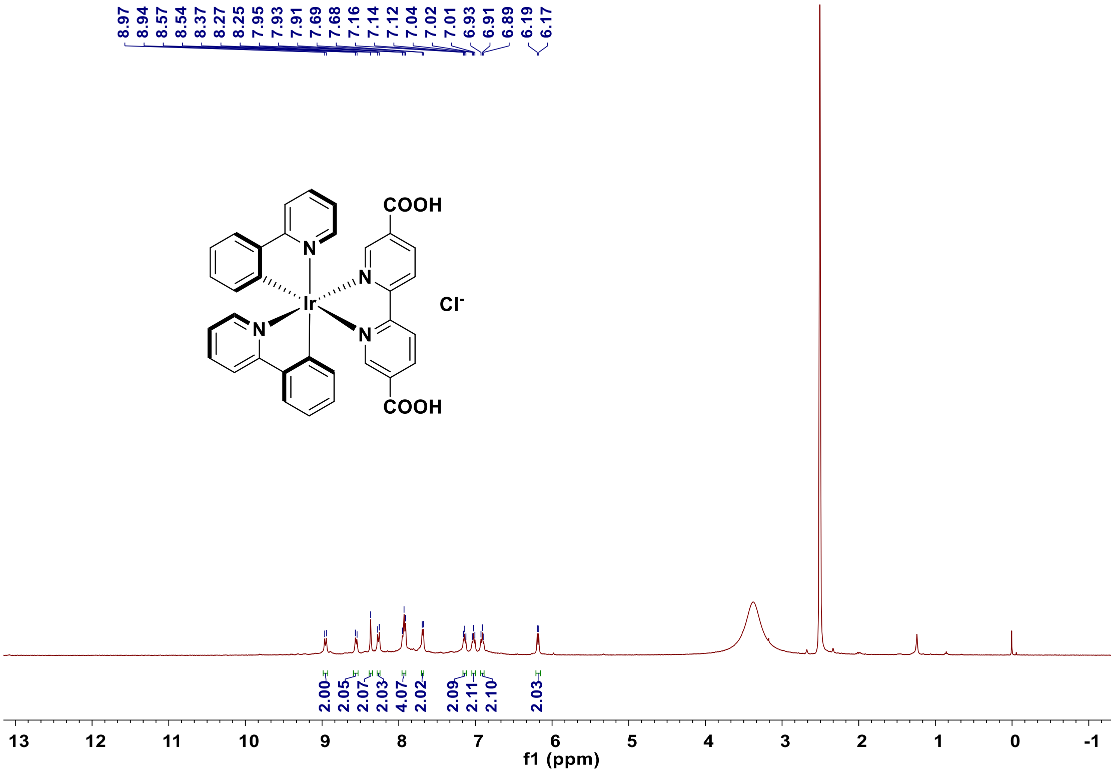

Supplementary Figure 1. ^{1}H NMR spectrum of **H$_2$L**.

Synthesis and characterizations of UiO-67-Ir and UiO-67-PdX$_2$ (X = OAc, TFA).

Synthesis of UiO-67-Ir. UiO-67-Ir was synthesized through solvothermal reaction of $ZrCl_4$ (0.24 mmol, 55.5 mg), **H_2L** (0.02 mmol, 15 mg), bpy (0.18 mmol, 45 mg) and acetic acid (10.8 mmol, 618 μL) in a solvent of DMF (7.5 mL) at 120 °C for 24 h. Then, the reaction was cooled to room temperature at a rate of 20 °C/h. The product was centrifugated and washed with DMF and acetone for three times respectively. After that, the solid was immersed in fresh acetone for three days to exchange the high boiling-point DMF. The acetone was refreshed every other day during the period. Finally, the solid was heated at 50 °C under vacuum to remove the trapped solvents in the pores.

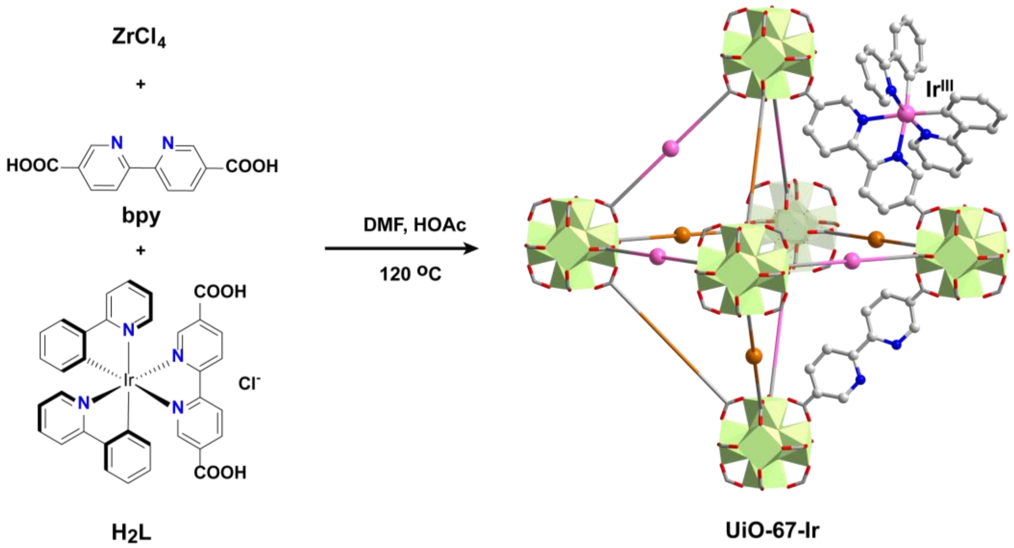

Supplementary Figure 2. Synthetic procedure for UiO-67-Ir.

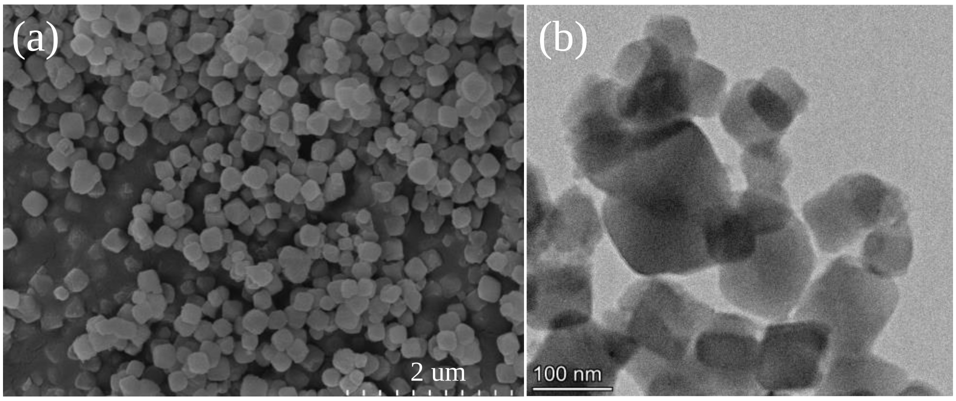

Supplementary Figure 3. (a) SEM and (b) TEM images of UiO-67-Ir.

^{1}H NMR of D_3PO_4 digested UiO-67-Ir. To determine the formula of UiO-67-Ir, 5 mg of dried UiO-67-Ir was digested in 50 μL D_3PO_4.and 1 mL DMSO-*d*6. The mixture was sonicated for 20 min to obtain the clear solution and then analyzed by ^{1}H NMR. The ratio between bpy/**L**/HOAc was determined to be about 7.14: 1: 1.91. The formula of UiO-67-Ir was thus determined to be $[Zr_6O_4(OH)_4bpy_{4.71}(L)_{0.66}(OAc)_{1.26}]$.

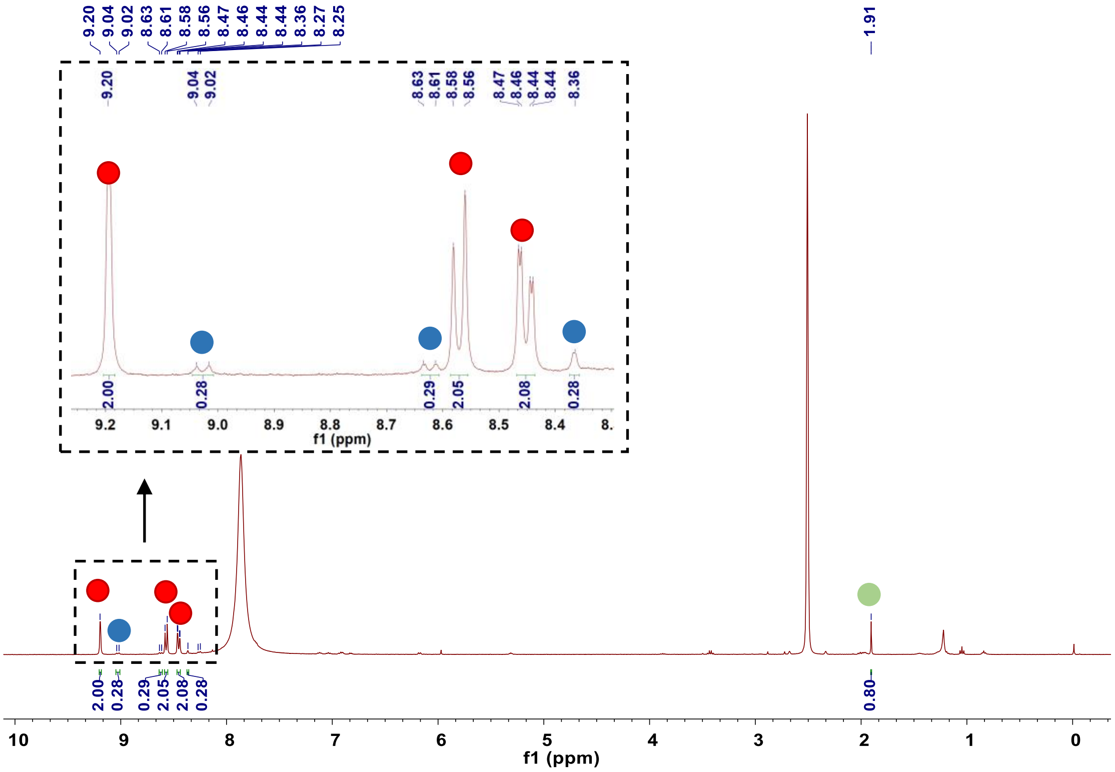

Supplementary Figure 4. ^{1}HNMR analyses of the D_3PO_4-digested UiO-67-Ir. Red, blue and green circles correspond to bpy, **L** and HOAc, respectively.

Thermogravimetric Analysis (TGA) of UiO-67-Ir. The first weight loss (4.83%) from 25 to 80 °C corresponds to the removal of adsorbed solvents in the MOF. The second weight loss (62.17%) from 80 - 900 °C corresponds to decomposition of UiO-67-Ir to metal oxides, consistent with a calculated weight loss of 62.85% based on the conversion of $Zr_6O_4(OH)_4bpy_{4.71}(L)_{0.66}(OAc)_{1.26}$ to 6 (ZrO_2) + 0.66 (IrO_2).

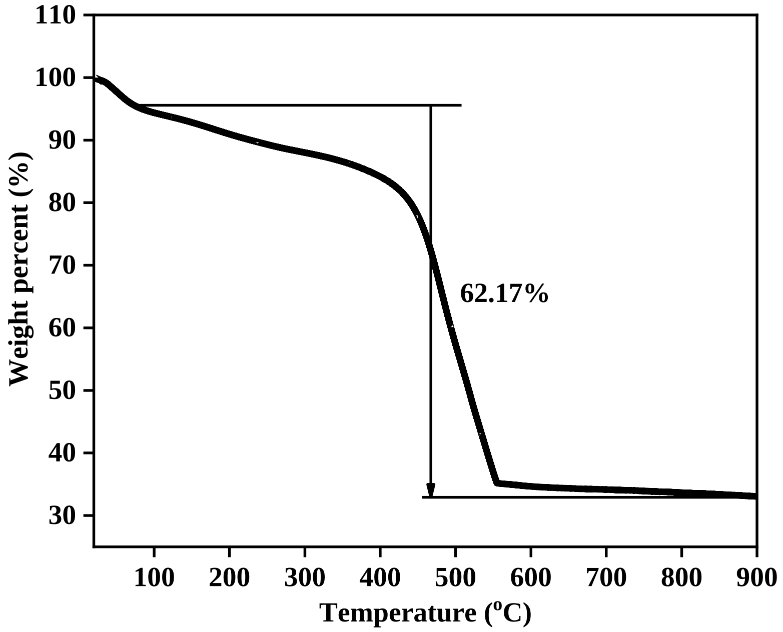

Supplementary Figure 5. TG curve of UiO-67-Ir.

Synthesis of UiO-67-PdX$_2$ (X = OAc, TFA). UiO-67-PdX$_2$ (X = OAc, TFA) were synthesized in two steps according to the previously reported literatures with slight modifications. $ZrCl_4$ (24.5 mg, 0.105 mmol), acetic acid (189 mg, 3.15 mmol), and bpy (26 mg, 0.105 mmol) were placed in a vial with 4 mL DMF. The solids were dispersed via sonication for ~10 mins, followed by incubation at 120 °C for 24 h. After cooling, solids were collected by centrifugation and the solvent was decanted. The solids were washed with DMF (2 × 10 mL), followed by soaking in methanol (MeOH) for 3 d, and the solution was exchanged with fresh MeOH (10 mL) every 24 h. After 3 d of soaking, the solids were collected via centrifugation and dried under vacuum. Activated UiO-67-bpy (50 mg) was dispersed in acetone (1.5 mL) and was sonicated for 30 min to achieve a homogeneous dispersion. Acetone solutions (1.5 mL) of $Pd(OAc)_2$ (10.2 mg, 0.048 mmol) or $Pd(TFA)_2$ (15.8 mg, 0.048 mmol) were added dropwise to the dispersion of UiO-67-bpy with vigorous stirring at room temperature. After that, the mixtures were centrifugated and the solids were washed with acetone for three times. The solids were immersed in acetone for 3 days, and acetone was refreshed every 24 h. After soaking, the solids were collected via centrifugation and dried under vacuum.

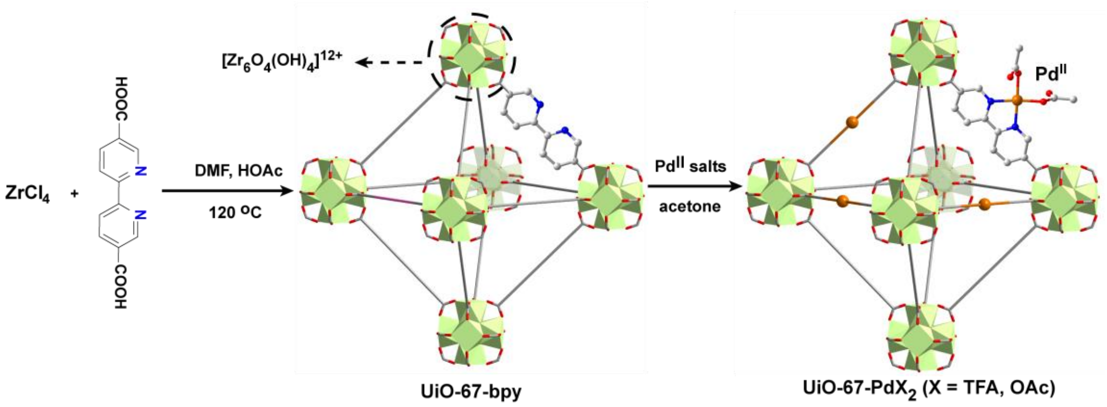

Supplementary Figure 6. Synthetic procedure for UiO-67-PdX_2 (X = OAc, TFA).

Synthesis and characterizations of UiO-67-Ir-PdX_2 (X = OAc, TFA).

Synthesis of UiO-67-Ir-PdX_2 (X = OAc, TFA). UiO-67-Ir (50 mg, 0.014 mmol based on Ir) was placed in 3 mL acetone of Pd(TFA)$_2$ (4.6 mg, 0.014 mmol) or Pd(OAc)$_2$ (3 mg, 0.014 mmol) solutions. The mixtures were sonicated for 10 min and then stirred at 50 ^{o}C for 24 h. After that, the mixtures were centrifugated and the solids were washed with acetone for three times. The solids were immersed in acetone for 3 days, and acetone was refreshed every other day during the period. After soaking, the solids were collected via centrifugation and dried at 50 ^{o}C under vacuum to obtain UiO-67-Ir-PdX_2 (X = OAc, TFA).

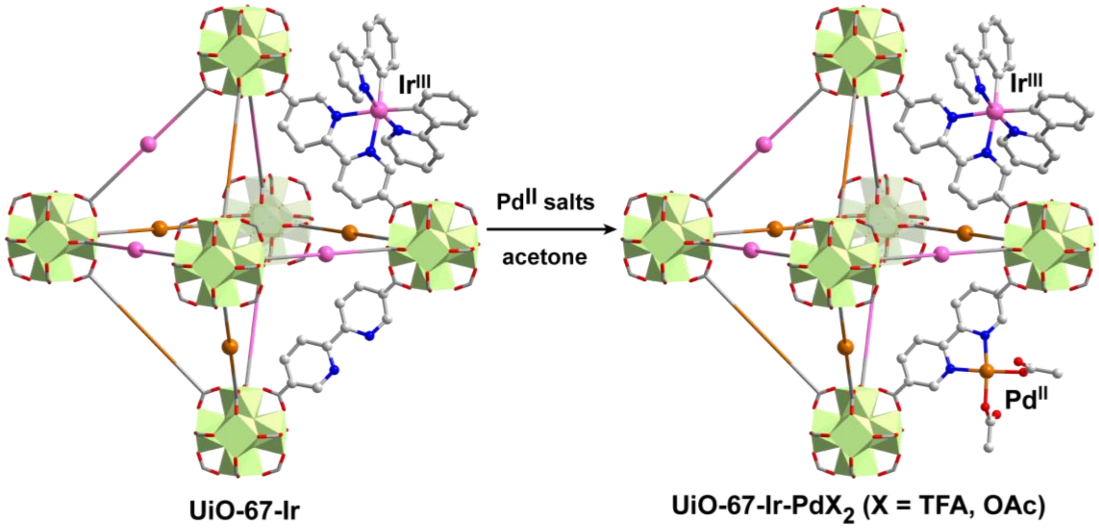

Supplementary Figure 7. Synthetic procedure for UiO-67-Ir-PdX_2 (X = OAc, TFA).

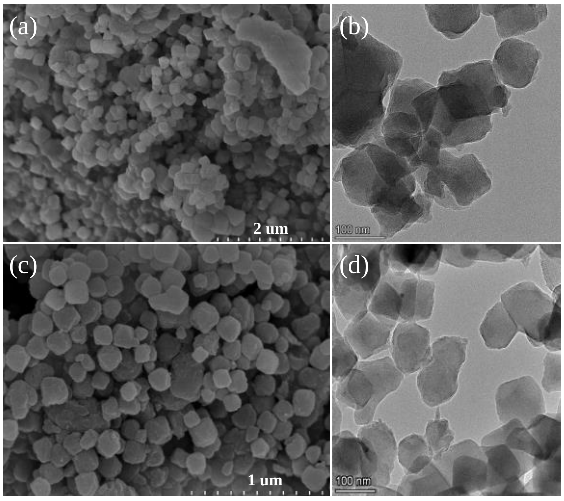

Supplementary Figure 8. SEM images of (a) UiO-67-Ir-Pd(OAc)$_2$, (c) UiO-67-Ir-Pd(TFA)$_2$ and TEM images of (b) UiO-67-Ir-Pd(OAc)$_2$, (d) UiO-67-Ir-Pd(TFA)$_2$.

N_2 adsorption isotherms of UiO-67-Ir and UiO-67-Ir-PdX$_2$ (X = OAc, TFA). The as-synthesized samples of UiO-67-Ir and UiO-67-Ir-PdX$_2$ (X = OAc, TFA) were firstly immersed in fresh acetone for 3 days to exchange the high-boiling point DMF. The exchanged MOF catalysts were then activated at 120 °C for 24 h before the adsorption experiments. The N_2 adsorption isotherms were recorded at 77 K and the BET surfaces areas of UiO-67-Ir, UiO-67-Ir-Pd(OAc)$_2$ and UiO-67-Ir-Pd(TFA)$_2$ were calculated to be 961, 621 and 526 m^2/g, respectively. The reduced N_2 adsorption amounts and calculated BET values of UiO-67-Ir-PdX$_2$ compared to UiO-67-Ir can be explained by the incorporation of Pd(OAc)$_2$ and Pd(TFA)$_2$ into the framework.

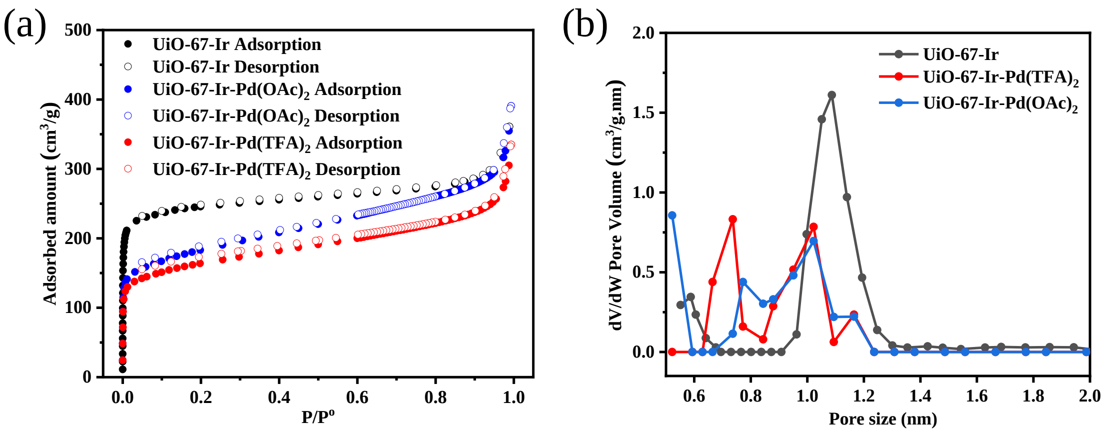

Supplementary Figure 9. N_2 adsorption isotherms (a) and pore size distributions (b) of UiO-67-Ir and UiO-67-Ir-PdX$_2$ (X = OAc, TFA) at 77 K.

The diffuse reflectance UV/vis and IR spectra of UiO-67-bpy, UiO-67-Ir and UiO-67-Ir-PdX$_2$ (X = OAc, TFA). The diffuse reflectance UV/vis spectra of UiO-67-Ir compared to UiO-67-bpy showed distinct adsorption in visible light region due to the incorporation of Ir^{III} complex PS within the framework. In specific, the absorption centered at about 400 nm can be assigned to spin-allowed metal-to-ligand charge transfer (MLCT) transitions.[1-2] The absorption above 460 nm is attributed to the spin-forbidden singlet-to-triplet MLCT transitions as a consequence of the heavy-atom effect resulting in a strong spin–orbit coupling.[3] The incorporation of Pd^{II} salts within the framework did not change the overall UV/vis spectra.

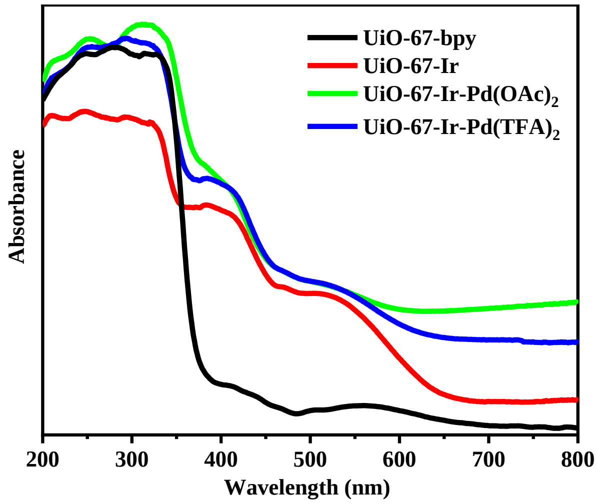

Supplementary Figure 10. The diffuse reflectance UV/vis spectra of UiO-67-bpy, UiO-67-Ir and UiO-67-Ir-PdX$_2$ (X = OAc, TFA).

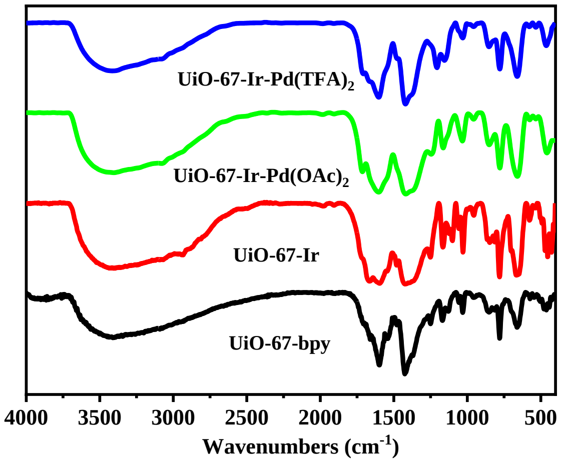

Supplementary Figure 11. The IR spectra of UiO-67-bpy, UiO-67-Ir and UiO-67-Ir-PdX$_2$ (X = OAc, TFA).

The XPS analyses of homogeneous complexes, UiO-67-Ir and UiO-67-Ir-PdX$_2$ (X = OAc, TFA).

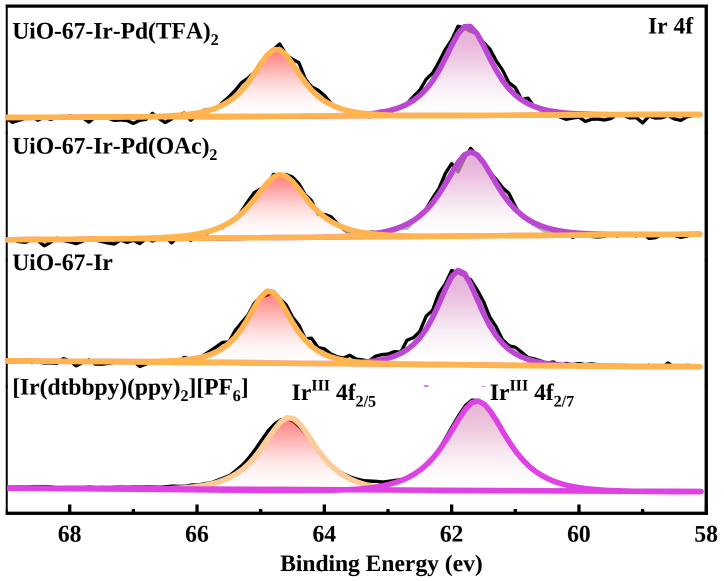

Supplementary Figure 12. The XPS spectra of the Ir 4f region of homogeneous Ir complex, UiO-67-Ir and UiO-67-Ir-PdX$_2$.

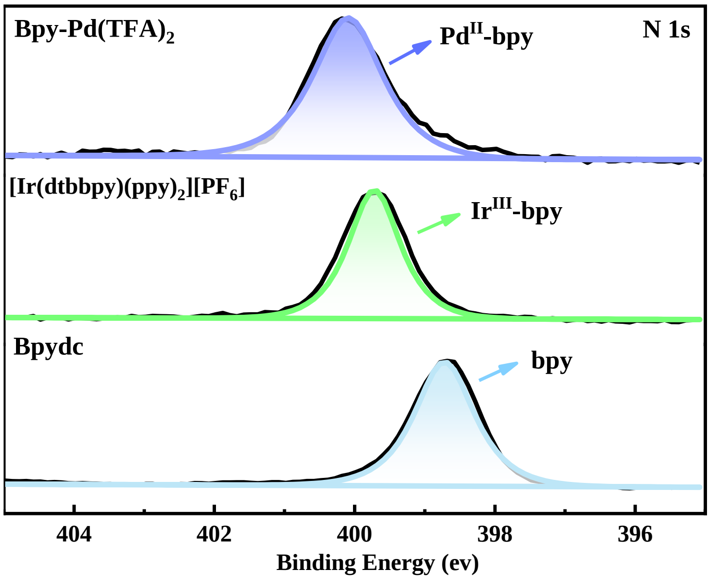

Supplementary Figure 13. The XPS spectra of the N 1s region of homogeneous Pd complex, Ir complex and Bpydc.

The X-ray absorption analyses of UiO-67-Ir-PdX$_2$ (X = OAc, TFA) and homogeneous complexes.

Data Collection of Pd. The Pd K-edge X-ray absorption fine structure (XAFS) measurements were performed at BL14W1 beamline in Shanghai Synchrotron

Radiation Facility (SSRF), China. The hard X-ray was monochromatized with Si (311) double-crystal monochromator and the XAFS data were collected in transmission mode in the energy range from -200 below to 1000 eV above the Pd K-edge.

EXAFS Fitting of Pd. The acquired EXAFS data were processed according to the standard procedures using the ATHENA module implemented in the IFEFFIT software packages. The k^2-weighted $\chi(k)$ data in the k-space ranging from 2.8-13.1 $Å^{-1}$ were Fourier transformed to real (R) space using a hanning windows (dk = 1.0 $Å^{-1}$) to separate the EXAFS contributions from different coordination shells. Quantitative EXAFS curve-fittings were carried out for the Fourier transformed $k^2\chi(k)$ in the R-space using the ARTEMIS module of IFEFFIT. Effective backscattering amplitudes F(k) and phase shifts Φ(k) of all fitting paths were calculated by the ab initio code FEFF8.0. During the fitting analysis, the amplitude reduction factor S_0^2 was fixed to the best-fit value of 0.82, which was determined from fitting the reference sample of Pd foil. As for Pd-bpy and UiO-67-Ir-PdX$_2$, the fitting was performed by including a single Pd-N/O shell, considering the difficulties in discriminating the N/O neighbors by EXAFS analysis. During curving fittings, the coordination number (N), Debye-Waller factors (σ^2), bond length (R) and energy shift (ΔE_0) were all treated as adjustable parameters.

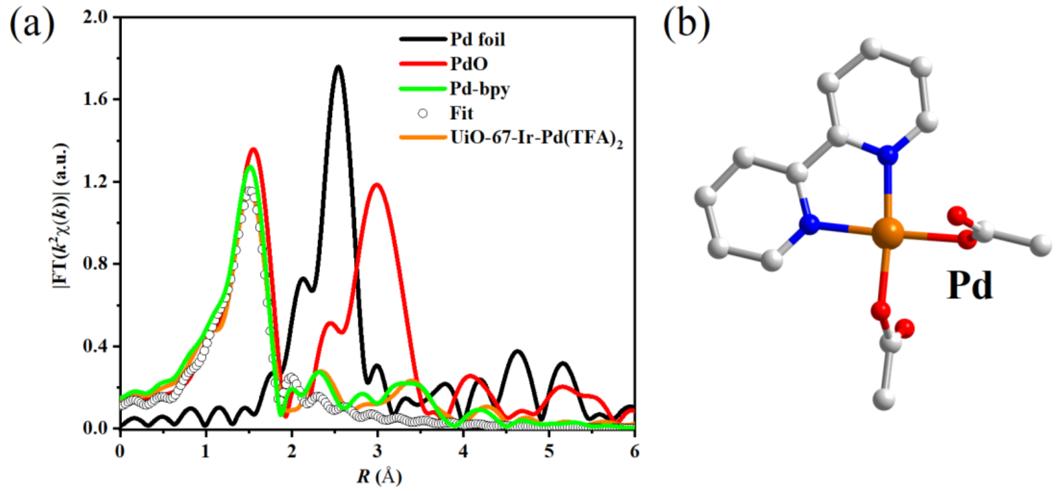

Supplementary Figure 14. (a) EXAFS spectra in R space at the Pd K-edge adsorption of Pd foil (black), PdO (red), Pd-bpy (green) and UiO-67-Ir-Pd(TFA)$_2$ (orange). (b) Molecular model of Pd complex.

Supplementary Table 1. EXAFS fitting parameters at the Pd K-edge for various samples.

Sample	Shell	N^a	R(Å)^b	σ^2×10^{-3}(Å^2)^c	ΔE_0 (eV)^d	R factor
Pd-bpy	Pd-N/O	3.6±0.3	1.98±0.01	2.1±0.9	0.2±0.5	0.002
UiO-67-Ir-Pd(OAc)$_2$	Pd-N/O	3.7±0.3	2.00±0.01	3.6±1.0	1.2±0.5	0.003
UiO-67-Ir-Pd(TFA)$_2$	Pd-N/O	3.6±0.4	1.99±0.01	3.2±1.2	0.5±0.8	0.005

$^a N$: coordination numbers; $^b R$: bond distance; $^c \sigma^2$: Debye-Waller factors; ^{d} ΔE_0: the inner potential correction. R factor: goodness of fit.

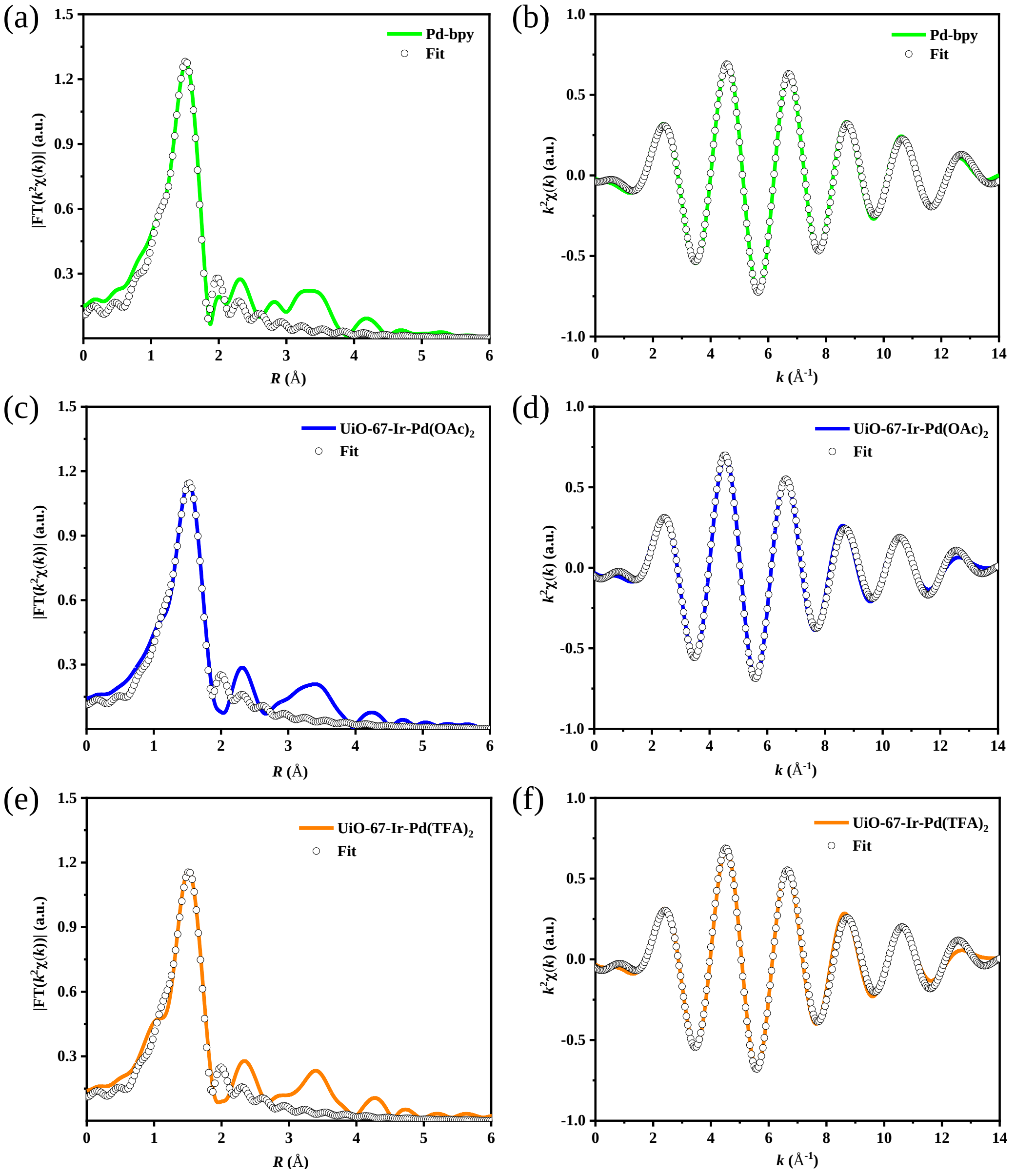

Supplementary Figure 15. EXAFS spectra in R space at the Pd K-edge adsorption of (a) Pd-bpy (c) UiO-67-Ir-Pd(OAc)$_2$ (e) UiO-67-Ir-Pd(TFA)$_2$. EXAFS spectra in K space at the Pd K-edge adsorption of (b) Pd-bpy (d) UiO-67-Ir-Pd(OAc)$_2$ (f) UiO-67-Ir-Pd(TFA)$_2$.

Data Collection of Ir. The XAFS spectra of [Ir(bpy)(ppy)$_2$](PF$_6$) and UiO-67-Ir-PdX$_2$ (X = OAc, TFA) at Ir L$_3$-edge were collected on the beamline BL07A1 in National Synchrotron Radiation Research Center (NSRRC). The radiation was monochromatized by a Si (111) double-crystal monochromator.

EXAFS Fitting of Ir. The obtained XAFS data was processed in Athena (version 0.9.26) for background, pre-edge line and post-edge line calibrations. Then Fourier transformed fitting was carried out in Artemis (version 0.9.26). The k^3 weighting, k-range of 3 - 10 Å^{-1} and R range of 1 - ~3 Å were used for the fitting of [Ir(bpy)(ppy)$_2$](PF$_6$) and UiO-67-Ir-PdX$_2$ (X = OAc, TFA). The four parameters, coordination number, bond length, Debye-Waller factor and E$_0$ shift (CN, R, σ^2 and ΔE_0) were fitted without anyone was fixed, constrained, or correlated.

Supplementary Table 2. EXAFS fitting parameters at the Ir L$_3$-edge for various samples.

Sample	Shell	CN^a	R(Å)^b	σ^2×10^{-3}(Å^2)^c	ΔE_0 (eV)^d	R factor
[Ir(bpy)(ppy)$_2$](PF$_6$)	Ir-C	2	2.01±0.01	2.5±0.5	9.8±0.3	0.006
	Ir-N	2	2.04±0.01			
	Ir-N	2	2.13±0.01			
	Ir-C	4	2.90±0.01	7.9±1.5	7.9±0.4	
	Ir-C	2	3.00±0.01			
	Ir-C	4	3.03±0.01			
	Ir-C	2	3.09±0.01			
UiO-67-Ir-Pd(OAc)$_2$	Ir-C	1	1.94±0.01	2.8±2.0	8.1±1.9	0.011
	Ir-C	1	1.99±0.01			
	Ir-N	2	2.05±0.01			
	Ir-N	2	2.12±0.01			
	Ir-C	3	2.78±0.02	4.0±3.3		
	Ir-C	1	2.83±0.02			
	Ir-C	2	2.89±0.02			
	Ir-C	2	2.96±0.02			
	Ir-C	1	3.00±0.02			
	Ir-C	3	3.04±0.02			
UiO-67-Ir-Pd(TFA)$_2$	Ir-C	1	1.97±0.02	4.3±2.9	10.3±3.4	0.008
	Ir-C	1	2.02±0.02			
	Ir-N	2	2.08±0.02			

	Ir-N	2	2.15±0.02			
	Ir-C	3	2.56±0.03	3.3±2.3		
	Ir-C	1	2.61±0.03			
	Ir-C	2	2.68±0.03			
	Ir-C	2	2.75±0.03			
	Ir-C	1	2.79±0.03			
	Ir-C	3	2.83±0.03			

[a]*CN*: coordination numbers; [b]*R*: bond distance; [c]σ^2: Debye-Waller factors; [d] ΔE_0: the inner potential correction. *R* factor: goodness of fit.

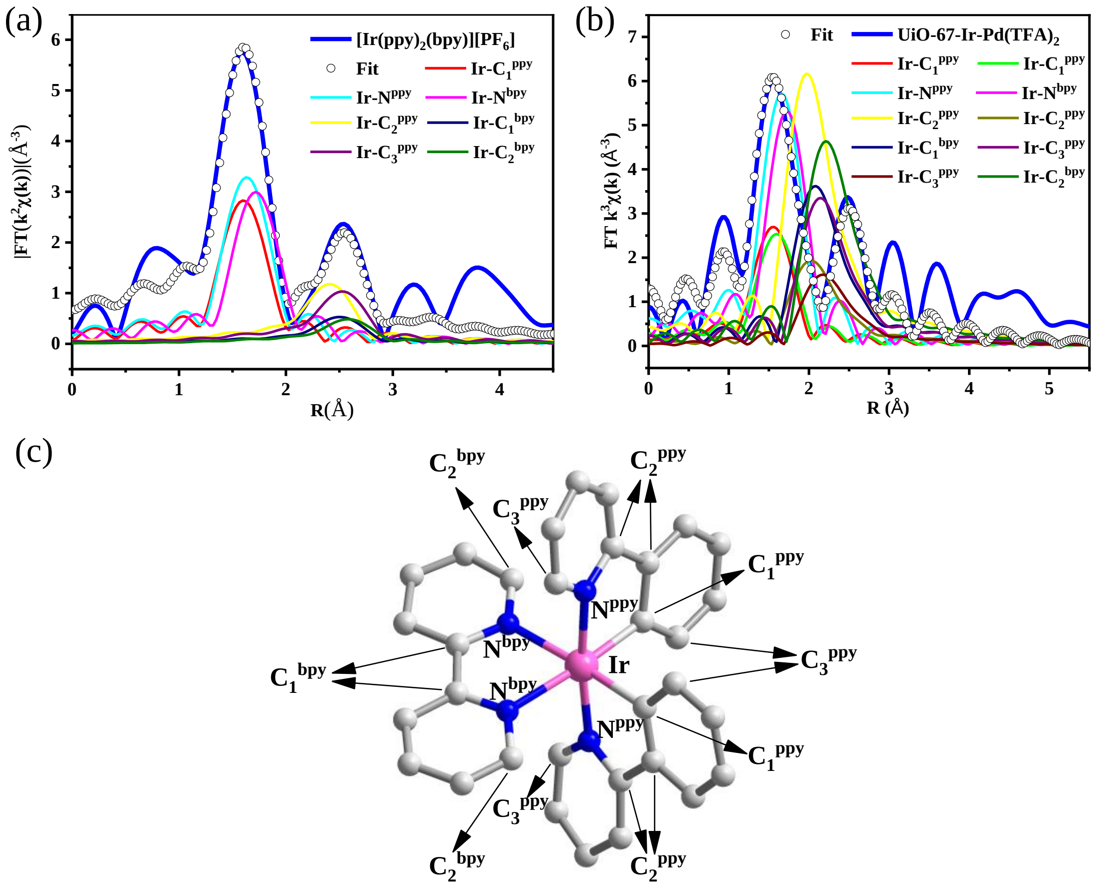

Supplementary Figure 16. EXAFS spectra (bule solid line) and fit (black circles) in R space at the Ir L_3-edge adsorption of (a) [Ir(bpy)(ppy)$_2$](PF$_6$) and (b) UiO-67-Ir-Pd(TFA)$_2$. (c) Molecular model of Ir species in UiO-67-Ir-PdX$_2$ (X = OAc, TFA), the atoms of each path for corresponding Ir-C/N bonds have been labeled.

General procedure for the homogenous Pd/photoredox-catalyzed decarboxylative coupling of allylic alcohols. A mixture of **1** (0.5 mmol), $Pd(TFA)_2$ (5 mol%, 8.3 mg) and $Ir(ppy)_3$ (1 mol%, 3.3 mg) was added in a 25 mL schlenk tube with a magnetic stir bar. The tube was outgassed completely and purged with O_2 for three cycles. Then, the toluene (2.85 mL) and DMSO (0.15 mL) solution of **2** (0.6 mmol) was injected into the mixture and the tube was charged with a O_2 balloon. Subsequently, the tube was placed into a constant temperature incubator to reduce the impact of the photo-induced heat. The mixture was vigorously stirred under a 40 W blue LED irradiation at room temperature for 48 h. After completion of the reaction, the mixture was extracted with ethyl acetate (3 × 10 mL). The combined ethyl acetate layer was then dried over sodium sulfate and concentrated under vacuum. The resulting crude product was purified by silica gel chromatography to afford the desired product.

Reaction optimization. With 2,6-dimethoxybenzoic acid (**1a**) and but-3-en-2-ol (**1b**) as the substrates, the optimal reaction condition can be obtained after considerable efforts with a yield of 96% (Supplementary Table 3, entry 1). Increasing or decreasing the dosage of $Ir(ppy)_3$ both gave inferior catalytic results (Supplementary Table 3, entries 2-3). Likewise, the substitution of $Ir(ppy)_3$ to other common PS such as $[Ru(bpy)_3](Cl)_2$ and $[Ru(bpy)_3](PF_6)_2$ lead to decreased yields (Supplementary Table 3, entries 4-5). Note that the loadings of Pd catalyst was also essential to this reaction (Supplementary Table 3, entry 6). When the reaction time was reduced to 24 h, an obvious decreased yield of 66% can be observed (Supplementary Table 3, entry 7). Control experiments implied that the reaction was quenched when performed in dark even at elevated temperature or in the absence of $Ir(ppy)_3$ (Supplementary Table 3, entries 8-9), suggesting the irreplaceable role of the photoexcited e-h pairs from the PS in the catalytic process. In addition, O_2 as the terminal oxidant was also found integral to this reaction (Supplementary Table 3, entry 10). Hence, the optimal reaction condition of the homogeneous Pd/photoredox-catalyzed decarboxylative

coupling of allylic alcohols is as follows: 5 mol% $Pd(TFA)_2$, 1 mol% $Ir(ppy)_3$ and O_2 at room temperature under blue LED irradiation for 48 h.

Supplementary Table 3. Reaction optimization of the homogeneous Pd/photoredox-catalyzed decarboxylative coupling between **1a** and **2a**.

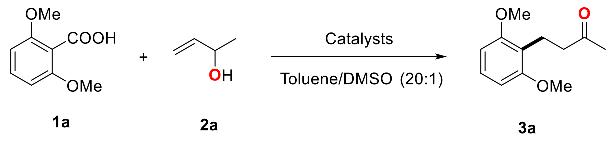

Entry	Variation from the standard conditions	Yield (%)[b]
1	none	96 (92)
2	0.5 mol% $Ir(ppy)_3$	70
3	2.5 mol% $Ir(ppy)_3$	61
4	$[Ru(bpy)_3](Cl)_2$ instead of $Ir(ppy)_3$	64
5	$[Ru(bpy)_3](PF_6)_2$ instead of $Ir(ppy)_3$	69
6	2.5 mol% $Pd(TFA)_2$	67
7	24 h	66
8	no light at 50 ^oC	n.d.
9	no $Ir(ppy)_3$	Trace
10	N_2	Trace

[a]Reaction Conditions: **1a** (0.5 mmol), **2a** (0.6 mmol), $Pd(TFA)_2$ (5 mol%), $Ir(ppy)_3$ (1 mol%), PhMe (2.85 mL), DMSO (0.15 mL), room temperature, 48 h, Schlenk tube charged with O_2; [b]Yields are determined by NMR with CH_2Br_2 as internal standard; isolated yield in the parentheses.

General procedure for the homogenous Pd/photoredox-catalyzed acetoxypalladation of various alkynes with alkenes. A mixture of **4** (0.5 mmol), $Pd(OAc)_2$ (10 mol%, 11.2 mg), KBr (40 mol%, 23.8 mg) and $Ir(ppy)_3$ (2.5 mol%, 8.2 mg) was added in a 25 mL schlenk tube with a magnetic stir bar. The tube was outgassed completely and purged with O_2 for three cycles. Then, the MeCN (2 mL) and HOAc (0.5 mL) solution of **5** (1 mmol) was injected into the mixture and the tube was charged with a O_2 balloon. Subsequently, the tube was placed into a constant temperature incubator to reduce the impact of the photo-induced heat. The mixture

was vigorously stirred under a 40 W blue LED irradiation at 60 °C for 48 h. After completion of the reaction, the mixture was firstly poured into the saturated $NaHCO_3$ solution and then extracted with ethyl acetate (3 × 10 mL). The combined ethyl acetate layer was then dried over sodium sulfate and concentrated under vacuum. The resulting crude product was purified by silica gel chromatography to afford the desired product.

Supplementary Table 4. Reaction optimization of the homogeneous Pd/photoredox-catalyzed acetoxypalladation/Heck coupling between **4a** and **5a**.

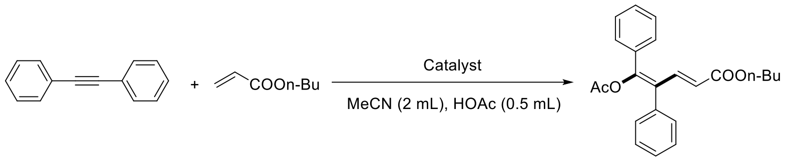

4a **5a** **6a**

Entry	Variation from the standard conditions	Yield (%)[b]
1	none	84 (80)
2	1 mol% $Ir(ppy)_3$	63
3	5 mol% $Ir(ppy)_3$	71
4	$[Ru(bpy)_3](Cl)_2$ instead of $Ir(ppy)_3$	43
5	$[Ru(bpy)_3](PF_6)_2$ instead of $Ir(ppy)_3$	47
6	5 mol% $Pd(OAc)_2$	60
7	24 h	55
8	r.t. instead of 60 °C	28
9	no light at 80 °C	9
10	no $Ir(ppy)_3$	7
11	N_2	<5

[a]Reaction Conditions: **4a** (0.5 mmol), **5a** (1 mmol), $Pd(OAc)_2$ (10 mol%), $Ir(ppy)_3$ (2.5 mol%), KBr (40 mol%), CH_3CN (2 mL), HOAc (0.5 mL), 60 °C, 48 h, schlenk tube charged with O_2; [b]Yields are determined by NMR with CH_2Br_2 as internal standard; isolated yield in the parentheses.

Reaction optimization. With 1,2-diphenylethyne (**4a**) and butyl acrylate (**5a**) as the starting materials, the optimal reaction condition can be obtained using 2.5 mol% of Ir(ppy)$_3$ at 60 ^{o}C for 48 h under blue LED illumination (Supplementary Table 4, entry 1). Whether an increase or decrease in the dosage of Ir(ppy)$_3$ results in slightly reduced yields (Supplementary Table 4, entries 2-3). The use of other Ru PS leads to obvious quenched catalytic activities (Supplementary Table 4, entries 4-5). Reducing the loading of Pd(OAc)$_2$ to 5 mol% also gave inferior catalytic result (Supplementary Table 4, entry 6). When the reaction time was reduced to 24 h, only moderate yield can be observed (Supplementary Table 4, entry 7). Notably, the temperature acts as a prominent factor to this system with a sharp decrease of the yield when the reaction was conducted at room temperature (Supplementary Table 4, entry 8). Control experiments confirmed the indispensable role of light, Ir(ppy)$_3$ and O_2 in this catalytic system (Supplementary Table 4, entries 9-11).

General procedure for the homogenous Pd/photoredox-catalyzed C−H alkenylation of 2-phenylphenol. A mixture of **7** (0.5 mmol), Pd(OAc)$_2$ (10 mol%, 11.2 mg), and Ir(ppy)$_3$ (1 mol%, 3.3 mg) was added in a 25 mL schlenk tube with a magnetic stir bar. The tube was outgassed completely and purged with O_2 for three cycles. Then, the DMSO (2 mL) solution of **8** (1 mmol) was injected into the mixture and the tube was charged with a O_2 balloon. Subsequently, the tube was placed into a constant temperature incubator to reduce the impact of the photo-induced heat. The mixture was vigorously stirred under a 40 W blue LED irradiation at 50 ^{o}C for 36 h. After completion of the reaction, the mixture was firstly poured into the aqueous solution and then extracted with ethyl acetate (3 × 10 mL). The combined ethyl acetate layer was then dried over sodium sulfate and concentrated under vacuum. The resulting crude product was purified by silica gel chromatography to afford the desired product.

Reaction optimization. With 2-phenylphenol (**7a**) and ethyl acrylate (**8a**) as the starting materials, the optimal reaction condition can be obtained using 1 mol% of Ir(ppy)$_3$ at 50 ^{o}C for 36 h under blue LED illumination (Supplementary Table 5, entry

1). Whether an increase or decrease in the dosage of $Ir(ppy)_3$ results in slightly reduced yields (Supplementary Table 5, entries 2-3). The use of other Ru PS leads to quenched catalytic activities (Supplementary Table 5, entries 4-5). Reducing the loading of $Pd(OAc)_2$ to 5 mol% also gave inferior catalytic result (Supplementary Table 5, entry 6). When the reaction time was reduced to 24 h, moderate yield can be observed (Supplementary Table 4, entry 7). Besides, the yield is decreased obviously when the reaction was conducted at room temperature (Supplementary Table 5, entry 8). Control experiments confirmed that the absence of light, $Ir(ppy)_3$ and O_2 led to reduced yields in this catalytic system (Supplementary Table 5, entries 9-11).

Supplementary Table 5. Reaction optimization of the homogeneous Pd/photoredox-catalyzed C−H alkenylation of 2-phenylphenol between **7a** and **8a**.

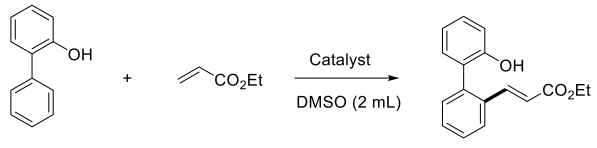

7a **8a** **9a**

Entry	Variation from the standard conditions	Yield (%)[b]
1	none	65 (63)
2	0.5 mol% $Ir(ppy)_3$	61
3	2.5 mol% $Ir(ppy)_3$	55
4	$[Ru(bpy)_3](Cl)_2$ instead of $Ir(ppy)_3$	47
5	$[Ru(bpy)_3](PF_6)_2$ instead of $Ir(ppy)_3$	41
6	5 mol% $Pd(OAc)_2$	40
7	24 h	53
8	r.t. instead of 50 ^{o}C	35
9	no light at 80 ^{o}C	43
10	no $Ir(ppy)_3$	45
11	N_2	11

[a]Reaction Conditions: **7a** (0.5 mmol), **8a** (1 mmol), $Pd(OAc)_2$ (10 mol%), $Ir(ppy)_3$ (1 mol%), DMSO (2 mL), 50 ^{o}C, 36 h, schlenk tube charged with O_2; [b]Yields are determined by NMR with CH_2Br_2 as internal standard; isolated yield in the parentheses.

General procedure for the MOFs based Pd/photoredox-catalyzed decarboxylative coupling of allylic alcohols. A mixture of **1** (0.5 mmol), UiO-67-Ir-Pd(TFA)$_2$ (0.5 mol% based on Pd) was added in a 25 mL schlenk tube with a magnetic stir bar. The tube was outgassed completely and purged with O_2 for three cycles. Then, the toluene (2.85 mL) and DMSO (0.15 mL) solution of **2** (0.6 mmol) was injected into the mixture and the tube was charged with a O_2 balloon. Subsequently, the tube was placed into a constant temperature incubator to reduce the impact of the photo-induced heat. The mixture was vigorously stirred under a 40 W blue LED irradiation at room temperature for 18 h. After completion of the reaction, the mixture was centrifugated to remove the solid phase, and the filtrate was extracted with ethyl acetate (3 × 10 mL). The combined ethyl acetate layer was then dried over sodium sulfate and concentrated under vacuum. The resulting crude product was purified by silica gel chromatography to afford the desired product. The recovered solid was thoroughly washed with acetone and immersed in acetone for 24 h. Then, the catalyst was dried at 50 ^oC under vacuum for reuse.

General procedure for the MOFs based Pd/photoredox-catalyzed acetoxypalladation of various alkynes with alkenes. A mixture of **4** (0.5 mmol), UiO-67-Ir-Pd(OAc)$_2$ (1 mol% based on Pd) and KBr (40 mol%, 23.8 mg) was added in a 25 mL schlenk tube with a magnetic stir bar. The tube was outgassed completely and purged with O_2 for three cycles. Then, the MeCN (2 mL) and HOAc (0.5 mL) solution of **5** (1 mmol) was injected into the mixture and the tube was charged with a O_2 balloon. Subsequently, the tube was placed into a constant temperature incubator to reduce the impact of the photo-induced heat. The mixture was vigorously stirred under a 40 W blue LED irradiation at 60 ^oC for 24 h. After completion of the reaction, the mixture was centrifugated to remove the solid phase. The filtrate was poured into the saturated $NaHCO_3$ solution and then extracted with ethyl acetate (3 × 10 mL). The combined ethyl acetate layer was then dried over sodium sulfate and concentrated under vacuum. The resulting crude product was purified by silica gel chromatography to afford the desired product. The recovered solid was thoroughly washed with

acetone and immersed in acetone for 24 h. Then, the catalyst was dried at 50 °C under vacuum for reuse.

Supplementary Table 6. Comparison of the homogeneous Pd/O_2, Pd/stoichiometric-oxidant, Pd/photoredox and MOFs based Pd/photoredox systems for the acetoxypalladation reaction of alkyne with alkene between **4a** and **5a**.[a]

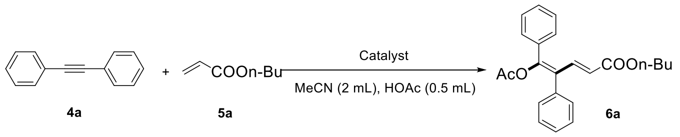

Entry	Catalytic system	t (h)	T (°C)	Yield (%)[b]	TON[c]	TOF[d]
Homogeneous Pd/O_2 system						
1	$Pd(OAc)_2$ (10 mol%)/O_2 (1 atm)	24	80	n.d.	-	-
Homogeneous Pd/stoichiometric-oxidants system						
2	$Pd(OAc)_2$ (10 mol%)/$Cu(OAc)_2$ (40 mol%)	24	80	89	8.9	0.37
Homogeneous Pd/photoredox system[e]						
3	$Pd(OAc)_2$ (10 mol%)/$Ir(ppy)_3$ (2.5 mol%)	48	60	84	8.4	0.18
4	$Pd(OAc)_2$ (1 mol%)/$Ir(ppy)_3$ (2.5 mol%)	48	60	24	24	0.5
5	$Pd(OAc)_2$ (10 mol%)/$Ir(ppy)_3$ (2.5 mol%)	24	60	55	5.5	0.23
MOFs based Pd/photoredox system[e]						
6	UiO-67-Ir-$Pd(OAc)_2$ [Pd (1 mol%)]	24	60	82	82	3.42
7	UiO-67-Ir-$Pd(OAc)_2$ [Pd (1 mol%)]	18	60	62	62	3.44
8[f]	UiO-67-Ir-$Pd(OAc)_2$ [Pd (1 mol%)]	24	80	11	11	0.46
9[g]	UiO-67-Ir-$Pd(OAc)_2$ [Pd (1 mol%)]	24	60	<5	-	-
10	UiO-67-$Pd(OAc)_2$ [Pd (1 mol%)]	24	60	8	8	0.33
11	UiO-67-Ir [Ir (0.5 mol%)]	18	25	n.d.	-	-
12	UiO-67-bpy (2 mol%) /$Pd(OAc)_2$ (10 mol%)/$Ir(ppy)_3$ (2.5 mol%)	24	60	54	5.4	0.23

[a]Reaction Conditions: **4a** (0.5 mmol), **5a** (1 mmol), KBr (40 mol%), solvent (2.5 mL). [b]Yields are determined by NMR with CH_2Br_2 as internal standard. [c]TON (turnover number): moles of product per mole of catalyst [d]TOF (turnover frequency): moles of product per mole of catalyst per hour. [e]O_2 atmosphere (1 atm), blue LED (40 W). [f]In dark. [g]under N_2.

Acetoxypalladation/coupling reactions catalyzed UiO-67-Ir-Pd(OAc)2. The reaction between **4a** and **5a** in the presence of UiO-67-Ir-Pd(OAc)2 showed remarkably superior catalytic performances for this reaction with low catalyst consumption, high catalyst stability and recyclability compared to the homogeneous systems (Supplementary Fig. 17).

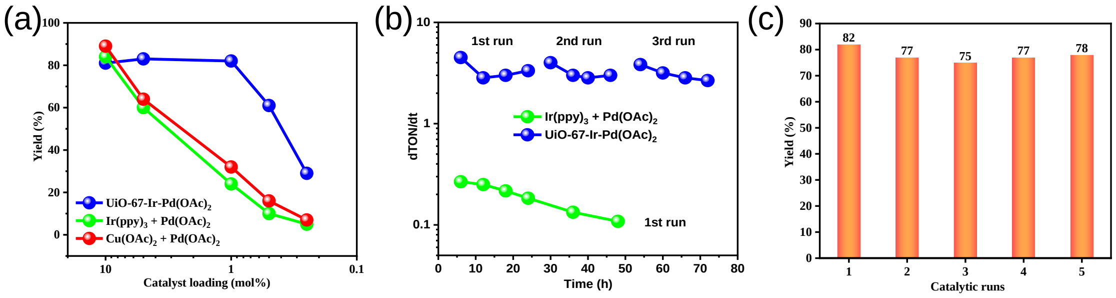

Supplementary Figure 17. (a) Plots of yields for **6a** *vs* Pd^{II} catalyst loading (b) the kinetic rates of of UiO-67-Pd(OAc)2 and homogeneous systems. (c) recyclability tests of UiO-67-Pd(OAc)2.

General procedure for the MOFs based Pd/photoredox-catalyzed C−H alkenylation of 2-phenylphenol. A mixture of **7** (0.5 mmol) and UiO-67-Ir-Pd(OAc)2 (0.5 mol% based on Pd) was added in a 25 mL schlenk tube with a magnetic stir bar. The tube was outgassed completely and purged with O_2 for three cycles. Then, the DMSO (2 mL) solution of **8** (1 mmol) was injected into the mixture and the tube was charged with a O_2 balloon. Subsequently, the tube was placed into a constant temperature incubator to reduce the impact of the photo-induced heat. The mixture was vigorously stirred under a 40 W blue LED irradiation at 50 ^{o}C for 12 h. After completion of the reaction, the mixture was centrifugated to remove the solid phase. The filtrate was poured into the aquous solution and then extracted with ethyl acetate (3 × 10 mL). The combined ethyl acetate layer was then dried over sodium sulfate and concentrated under vacuum. The resulting crude product was purified by silica gel chromatography to afford the desired product. The recovered solid was thoroughly washed with acetone and immersed in acetone for 24 h. Then, the catalyst was dried at 50 ^{o}C under vacuum for reuse.

Supplementary Table 7. Comparison of the homogeneous Pd/O_2, Pd/stoichiometric-oxidant, Pd/photoredox and MOFs based Pd/photoredox systems for the C−H alkenylation of 2-phenylphenol between **7a** and **8a**.[a]

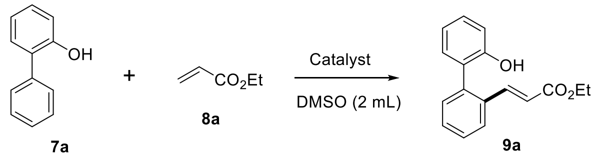

Entry	Catalytic system	t (h)	T (°C)	Yield (%)[b]	TON[c]	TOF[d]
Homogeneous Pd/O_2 system						
1	$Pd(OAc)_2$ (10 mol%)/O_2 (1 atm)	12	80	45	4.5	0.38
Homogeneous Pd/stoichiometric-oxidants system						
2	$Pd(OAc)_2$ (10 mol%)/BQ (100 mol%)	12	80	72	7.2	0.6
Homogeneous Pd/photoredox system[e]						
3	$Pd(OAc)_2$ (10 mol%)/Ir(ppy)$_3$ (1 mol%)	36	50	65	6.5	0.18
4	$Pd(OAc)_2$ (0.5 mol%)/Ir(ppy)$_3$ (1 mol%)	36	50	10	20	0.56
5	$Pd(OAc)_2$ (10 mol%)/Ir(ppy)$_3$ (1 mol%)	12	50	35	3.5	0.29
MOFs based Pd/photoredox system[e]						
6	UiO-67-Ir-Pd(OAc)$_2$ [Pd (0.5 mol%)]	12	50	80	160	13.3
7	UiO-67-Ir-Pd(OAc)$_2$ [Pd (0.5 mol%)]	6	50	45	90	15
8[f]	UiO-67-Ir-Pd(OAc)$_2$ [Pd (0.5 mol%)]	12	80	11	22	1.83
9[g]	UiO-67-Ir-Pd(OAc)$_2$ [Pd (0.5 mol%)]	12	50	13	26	2.17
10	UiO-67-Pd(OAc)$_2$ [Pd (0.5 mol%)]	12	50	8	16	1.33
11	UiO-67-Ir [Ir (0.5 mol%)]	12	50	n.d.	-	-
12	UiO-67-bpy (2 mol%) /Pd(OAc)$_2$ (10 mol%)/Ir(ppy)$_3$ (1 mol%)	12	50	37	3.7	0.3

[a]Reaction Conditions: **4a** (0.5 mmol), **5a** (1 mmol), KBr (40 mol%), solvent (2.5 mL). [b]Yields are determined by NMR with CH_2Br_2 as internal standard. [c]TON (turnover number): moles of product per mole of catalyst [d]TOF (turnover frequency): moles of product per mole of catalyst per hour. [e]O_2 atmosphere (1 atm), blue LED (40 W). [f]In dark. [g]under N_2.

C−H alkenylation of 2-phenylphenol catalyzed UiO-67-Ir-Pd(OAc)$_2$. The reaction between **7a** and **8a** in the presence of UiO-67-Ir-Pd(OAc)$_2$ showed remarkably superior catalytic performances for this reaction with low catalyst consumption, high

catalyst stability and recyclability compared to the homogeneous systems (Supplementary Fig. 18).

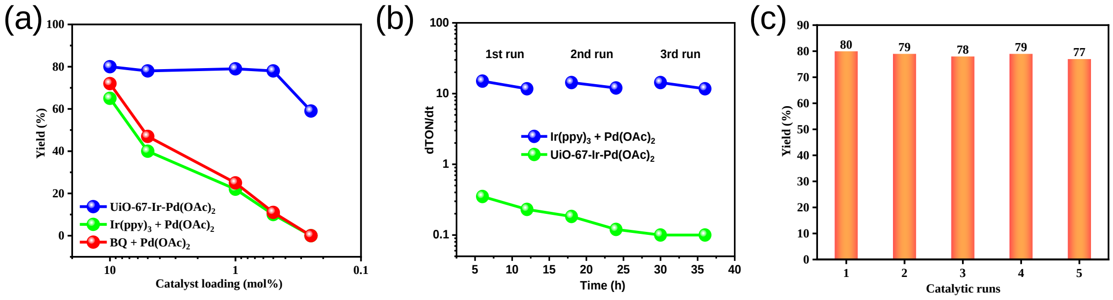

Supplementary Figure 18. (a) Plots of yields for **9a** *vs* Pd^{II} catalyst loading (b) the kinetic rates of of UiO-67-Pd(OAc)$_2$ and homogeneous systems. (c) recyclability tests of UiO-67-Pd(OAc)$_2$.

Characterizations of UiO-67-Ir-PdX$_2$ (X = OAc, TFA) after catalysis.

PXRD patterns.

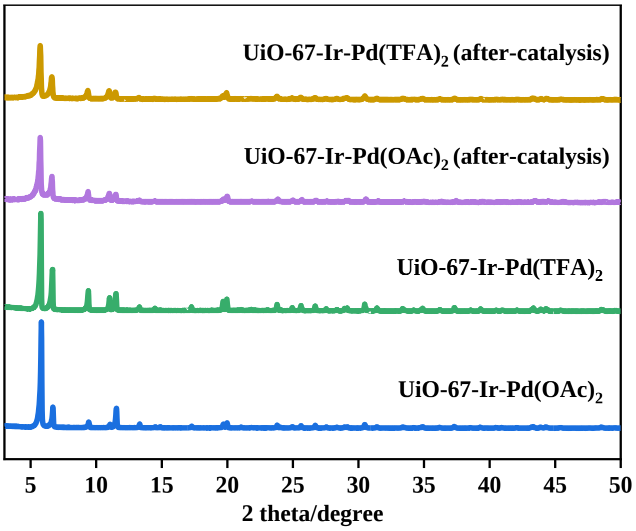

Supplementary Figure 19. The PXRD patterns of UiO-67-Ir-PdX$_2$ (X = OAc, TFA) and recovered UiO-67-Ir-PdX$_2$ (X = OAc, TFA) after catalysis.

XPS analyses.

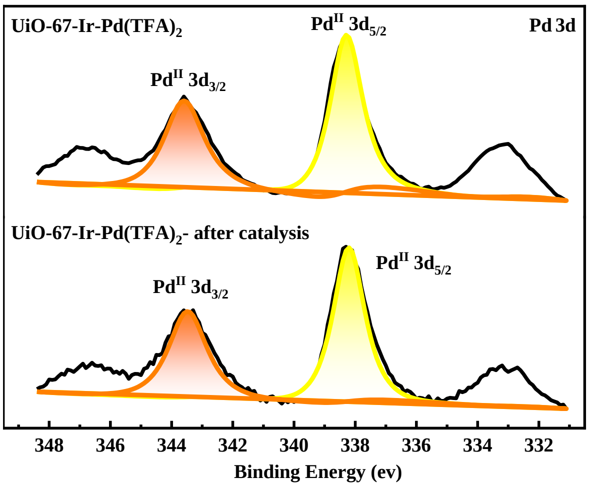

Supplementary Figure 20. XPS spectra of the Pd 3d region for UiO-67-Ir-Pd(TFA)$_2$.

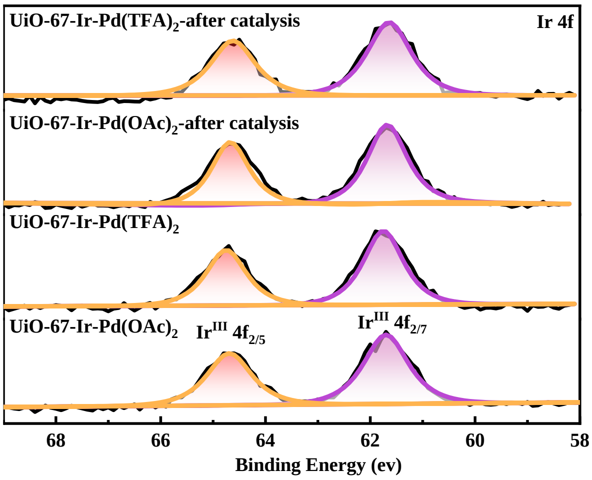

Supplementary Figure 21. XPS spectra of the Ir 4f region for UiO-67-Ir-PdX$_2$ (X = OAc, TFA) after catalysis.

TEM and mapping images.

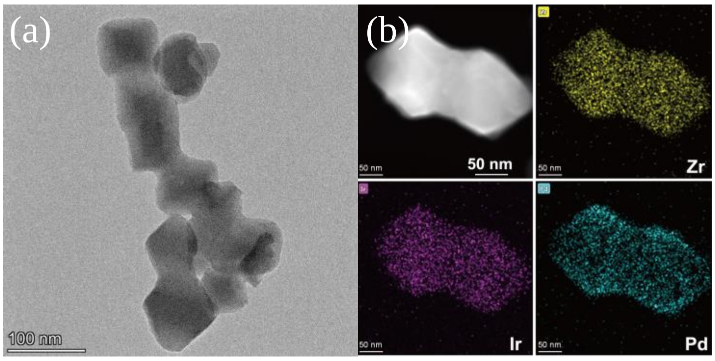

Supplementary Figure 22. (a) TEM images and (b) elemental mapping images of UiO-67-Ir-Pd(TFA)$_2$ after five consecutive runs under optimal reaction conditions.

XPS and TEM images of UiO-67-Ir-Pd(TFA)$_2$ after catalysis without light. When the reaction was performed without light irradiation, the XPS experiment of the recovered catalysts in Pd region was conducted, and the result showed distinct XPS characteristic peaks of Pd^0 (Supplementary Fig. 23a). While under the same reaction conditions, no characteristic peak of Ir^0 was found (Supplementary Fig. 23b). According to these XPS results, the NPs in Supplementary Fig. 23c can be confirmed to be Pd NPs.

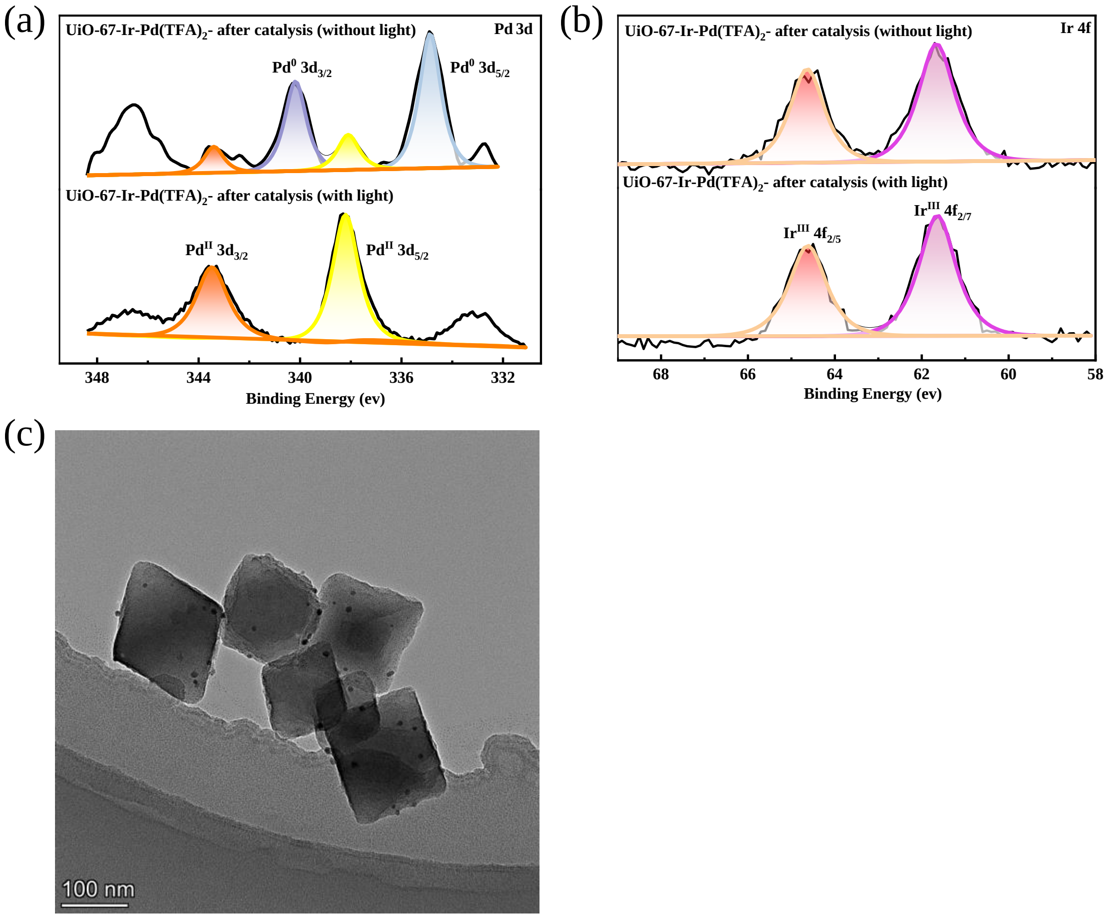

Supplementary Figure 23. XPS spectra of (a) the Pd 3d region (b) the Ir 4f region for UiO-67-Ir-Pd(TFA)$_2$ after catalysis without light irradiation. (c) TEM images of UiO-67-Ir-Pd(TFA)$_2$ after catalysis without light irradiation.

Femtosecond transient absorption (fs-TA) spectroscopy.

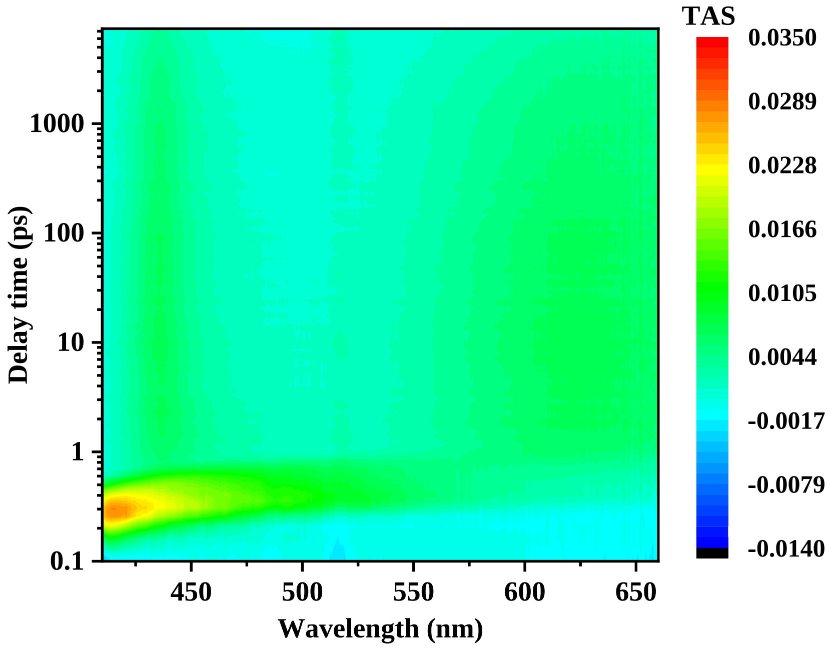

Supplementary Figure 24. Contour plot of fs-TA spectrum for UiO-67-Ir.

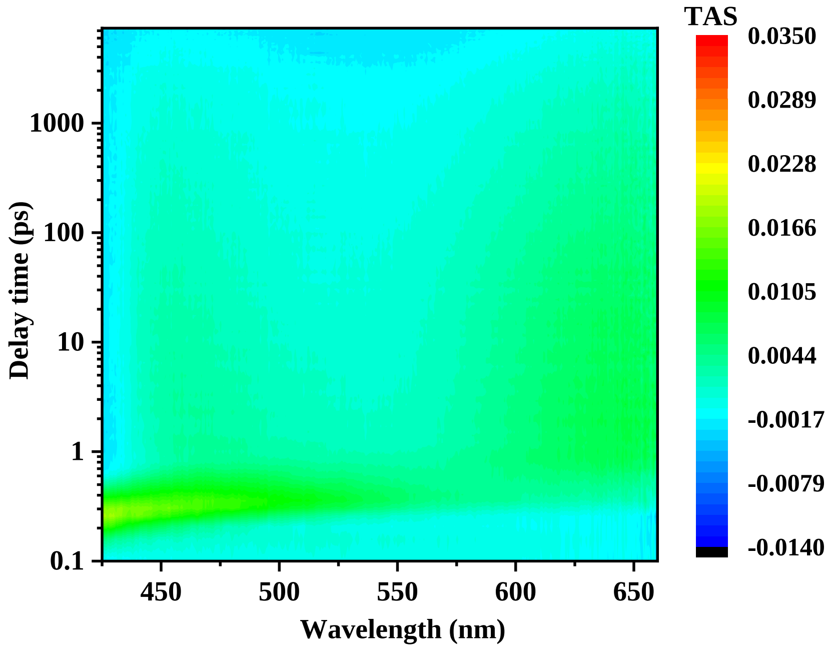

Supplementary Figure 25. Contour plot of fs-TA spectrum for UiO-67-Ir-PdNPs.

Photoluminescence (PL) experiments.

Preparation of UiO-67-Ir-PdNPs with different Pd NPs loadings. To a 4 mL glass vial was added 1 mL acetone suspension of UiO-67-Ir (6.0 μmol based on Ir) and $Pd(TFA)_2$ of different amount (0.25, 0.5, 0.75, and 1.0 eq of Ir). The reaction mixture was stirred at 50 ^oC for 24 h. The solid was collected by centrifugation and washed

with acetone for three times and then immersed in fresh acetone for 3 days. The acetone was refreshed every other day during the period. Subsequently, the solid was heated at 50 °C under vacuum to remove the trapped solvents in the pores. Finally, the samples of UiO-67-Ir-Pd(TFA)$_2$ with different Pd/Ir ratio were added in Pd-catalyzed decarboxylative coupling of allylic alcohols under the optimal reaction condition but without light to obtain UiO-67-Ir-PdNPs. The ratio between Pd and Ir for these recovered UiO-67-Ir-PdNPs was determined by ICP-MS to be 0.23, 0.54, 0.77, 0.97, respectively.

Preparation of UiO-67-PdNPs. The synthesis of UiO-67-PdNPs was according to the previously reported method.[4] $ZrCl_4$ (70.0 mg), (5,5'-dicarboxy-2,2'-bipyridine-) palladium(II)] dichloride (4.2 mg) and H_2bpdc (H_2bpdc, para-biphenyldicarboxylic acid) (70.24 mg) were dispersed in DMF (10 mL), sealed in a 20 mL vial at 100 °C for 36 h. Then, the reaction was cooled to room temperature at a rate of 20 °C/h. The produced powders were isolated by centrifugation and dried at ambient temperature. Subsequently, the as synthesized sample was soaked in chloroform for three 18 hours periods at room temperature to remove DMF and ligand precursors, then filtered off and dried under vacuum at room temperature for 24 h. Finally, the as-synthesized sample was treated in the stream of H_2 at 250 °C for 4 h to obtain the UiO-67-PdNPs. The Pd NP loadings in UiO-67-PdNP was determined to be 1 wt%.

Preparation of poly(N-vinyl-2-pyrrolidone) (PVP) stabilized Pd NPs. The synthesis of PVP-PdNPs was according to the previously reported method.[5] In a round bottle, aqueous Na_2PdCl_4 solution (1.0×10^{-4} mol·cm^{-3}) was added to a preheated (363 K) aqueous mixture (30 cm^3) containing ascorbic acid (8.5×10^{-4} mol) and PVP (5.0×10^{-3} mol; PVP/Pd molar ratio = 10). The mixture was kept under stirring at 363 K for 3 h, and a solution of Pd NPs was obtained. The Pd NPs were cleaned up from the excess of PVP via flocculation with acetone (1/3 v/v solution/acetone), rinsed thoroughly with acetone, and redispersed in water attaining ca. 0.4 wt% Pd in the final solution.

Photoluminescence quenching of Ir(ppy)3. To a 4 mL cuvette was added 3 mL DMSO solution of Ir(ppy)3 (100 μM based on Ir). Different amounts of UiO-67-PdNPs or PVP-PdNPs were then dispersed into the solution to achieve the final concentration of 20, 40, 60, 80, and 100 μM (based on Pd), respectively. The luminescence intensity of Ir(ppy)3 in these suspensions were measured separately, as shown in Supplementary Fig. 26.

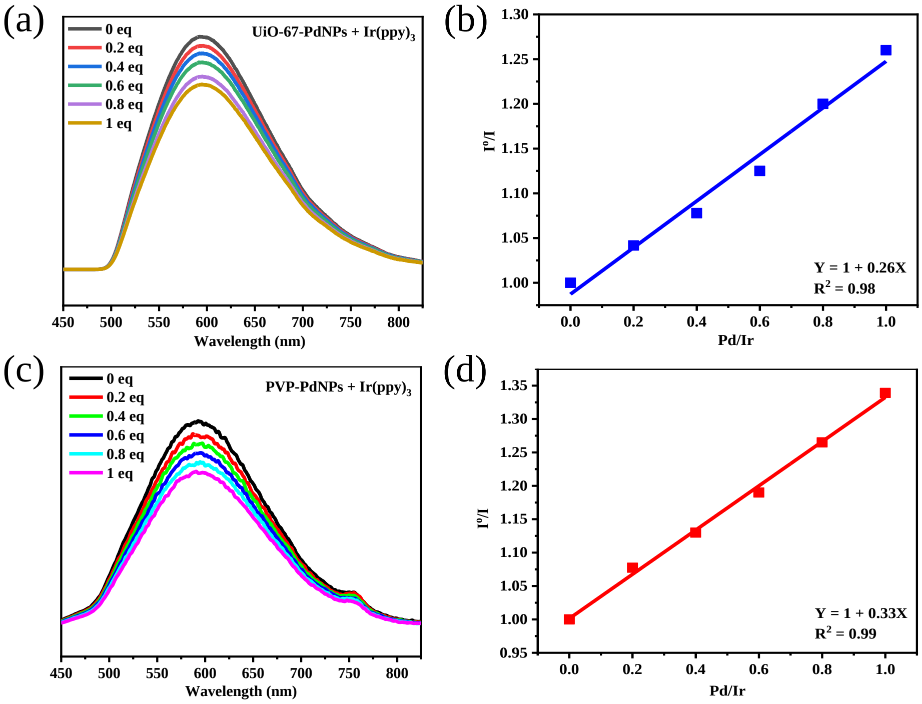

Supplementary Figure 26. Photoluminescence quenching curves of Ir(ppy)3 with different amounts of (a) UiO-67-PdNPs, (c) PVP-PdNPs. Fitting results of (b) UiO-67-PdNPs, (d) PVP-PdNPs.

Photoluminescence quenching of UiO-67-Ir-PdNPs. To a 4 mL cuvette was added 3 mL DMSO suspension of UiO-67-Ir-PdNPs (100 μM based on Ir) with different Pd NPs loadings. The luminescence intensity of UiO-67-Ir-PdNPs were measured separately, as shown in Supplementary Fig. 27.

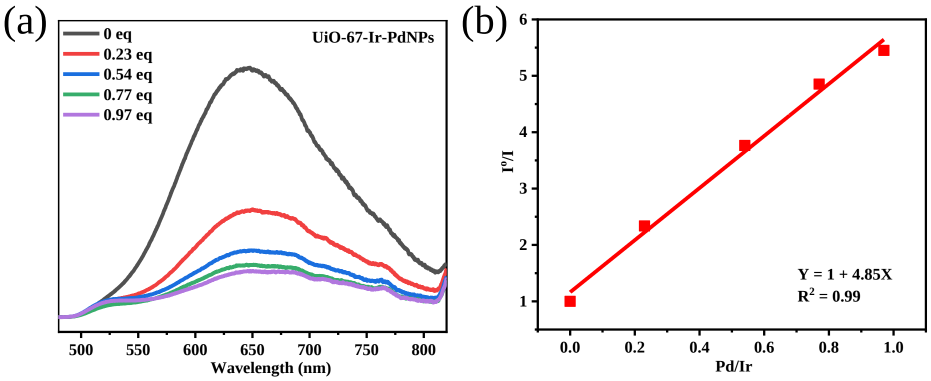

Supplementary Figure 27. (a) Photoluminescence quenching curves (b) fitting results of UiO-67-Ir-PdNPs with different Pd NPs loadings.

The Determination of Superoxide Radical Anions by EPR. 5,5-dimethyl-pyrroline-N-oxide (DMPO) was employed as the superoxide radical anion ($O_2^{-\bullet}$) sensitive trapping agent. As shown in Supplementary Fig. 28, the toluene solution of DMPO and UiO-67-Ir-Pd(TFA)$_2$ was performed under dark, leading to no signal was detected. In contrast, upon irradiation with blue LED, each irradiation was performed for 30 s for two consecutive irradiations, the enhanced signal intensity of DMPO-$O_2^{-\bullet}$ was observed, clearly confirming the production of $O_2^{-\bullet}$.

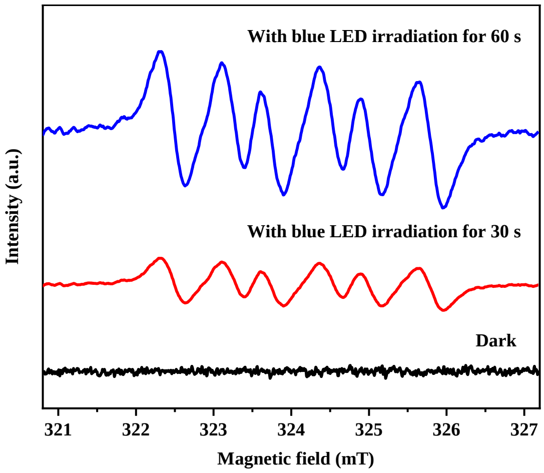

Supplementary Figure 28. EPR spectra of UiO-67-Ir-Pd(TFA)$_2$ for the detection of $O_2^{-\bullet}$ in dark or after illumination for 30 and 60 s.

Proposed mechanisms.

Pd-catalyzed decarboxylative coupling of allylic alcohols. The aromatic benzoic acid is firstly coordinated to Pd^{II} with the extrusion of CO_2. Then, the Heck-type insertion of the allyl alcohol to the arylpalladium complex occurs, affording the σ-allkylpalladium species. The selective β-H elimination of the σ-allkylpalladium species gives the enol complex, which subsequently transfer the hydrogen to the α-carbon atom by spontaneous insertion of $Pd^{II}H$. Either elimination from the a-OH or an anion-mediated reductive elimination is possible to afford the final product, accompanied by the generation of a Pd^0 species. Finally, the rapid SET process within MOF would occur between the *in situ* generated Pd^0 species and $[Ir^{III}]^*$, leading to the fast regeneration of active Pd^{II} catalyst and Ir^{II} species, which was reoxidized to Ir^{III} by O_2 to finish the photocatalytic cycle.

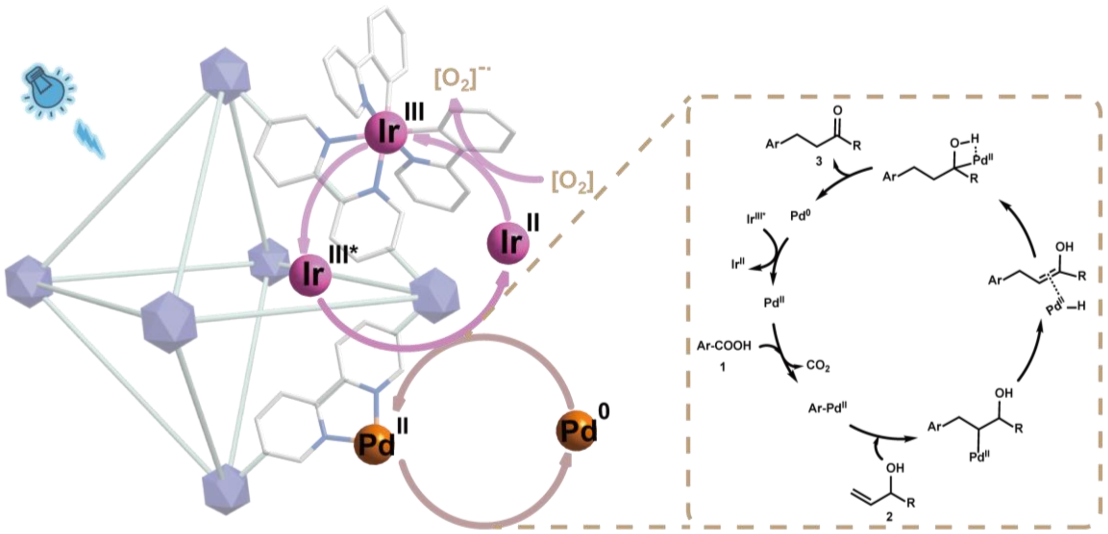

Supplementary Figure 29. Proposed mechanism for Pd-catalyzed decarboxylative coupling of allylic alcohols.

Pd-catalyzed acetoxypalladation of alkynes with alkenes. The reaction was initiated by the trans-acetoxypalladation of alkynes and (*E*)-vinylpalladium intermediate was formed. A subsequent Heck cross-coupling with alkenes, followed by further *β*-H elimination gave the targeted product and release the Pd^0 species. The fast SET process within MOF would occur between the *in situ* generated Pd^0 species and $[Ir^{III}]^*$, leading to the fast regeneration of active Pd^{II} catalyst and Ir^{II} , which was reoxidized to Ir^{III} by O_2 to finish the photocatalytic cycle.

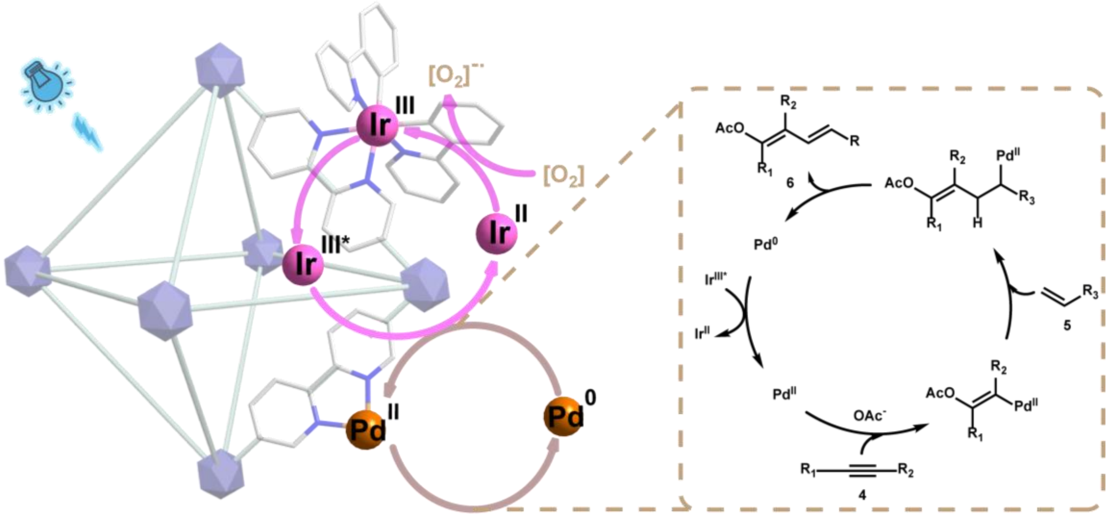

Supplementary Figure 30. Proposed mechanism for Pd-catalyzed acetoxypalladation of alkynes with alkenes.

^{1}H NMR and ^{13}C NMR spectra of the substrates in Pd-catalyzed decarboxylative coupling of allylic alcohols.

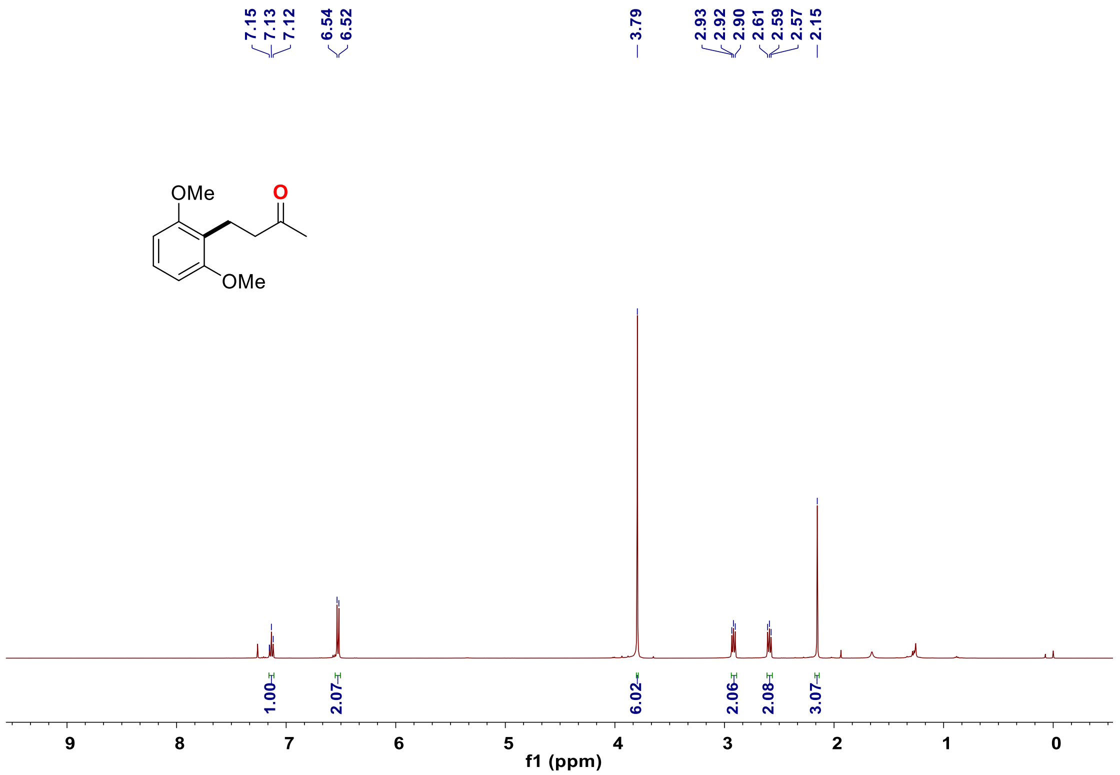

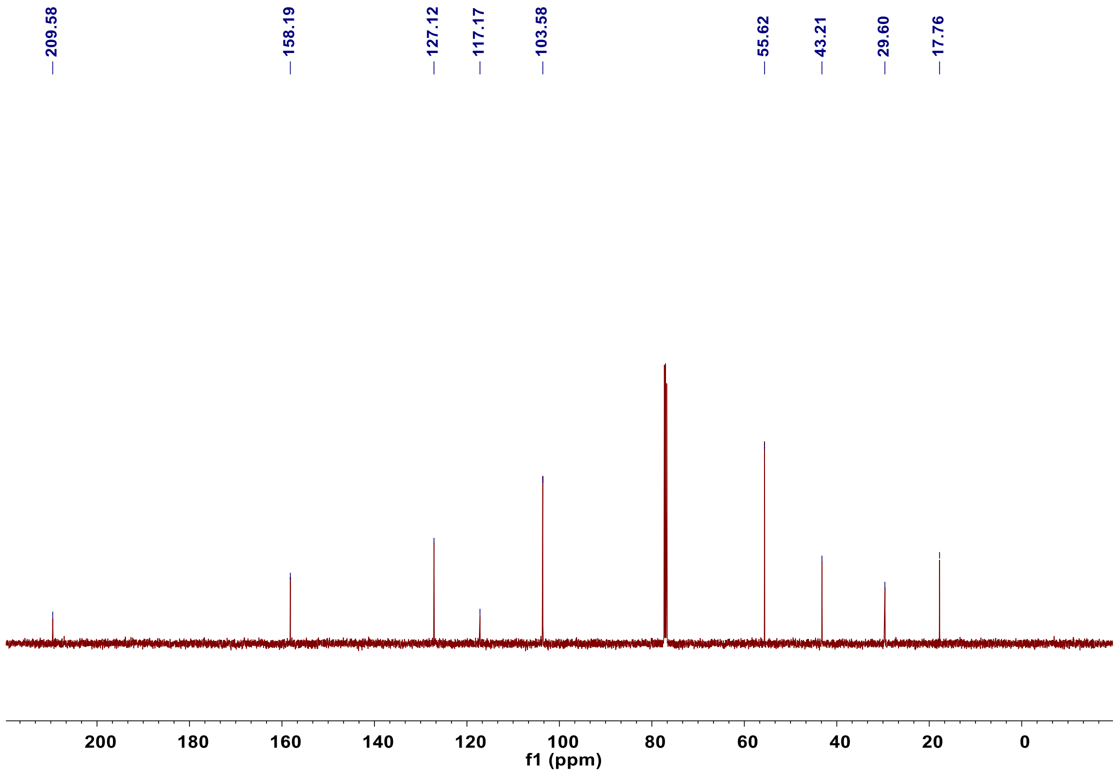

3a

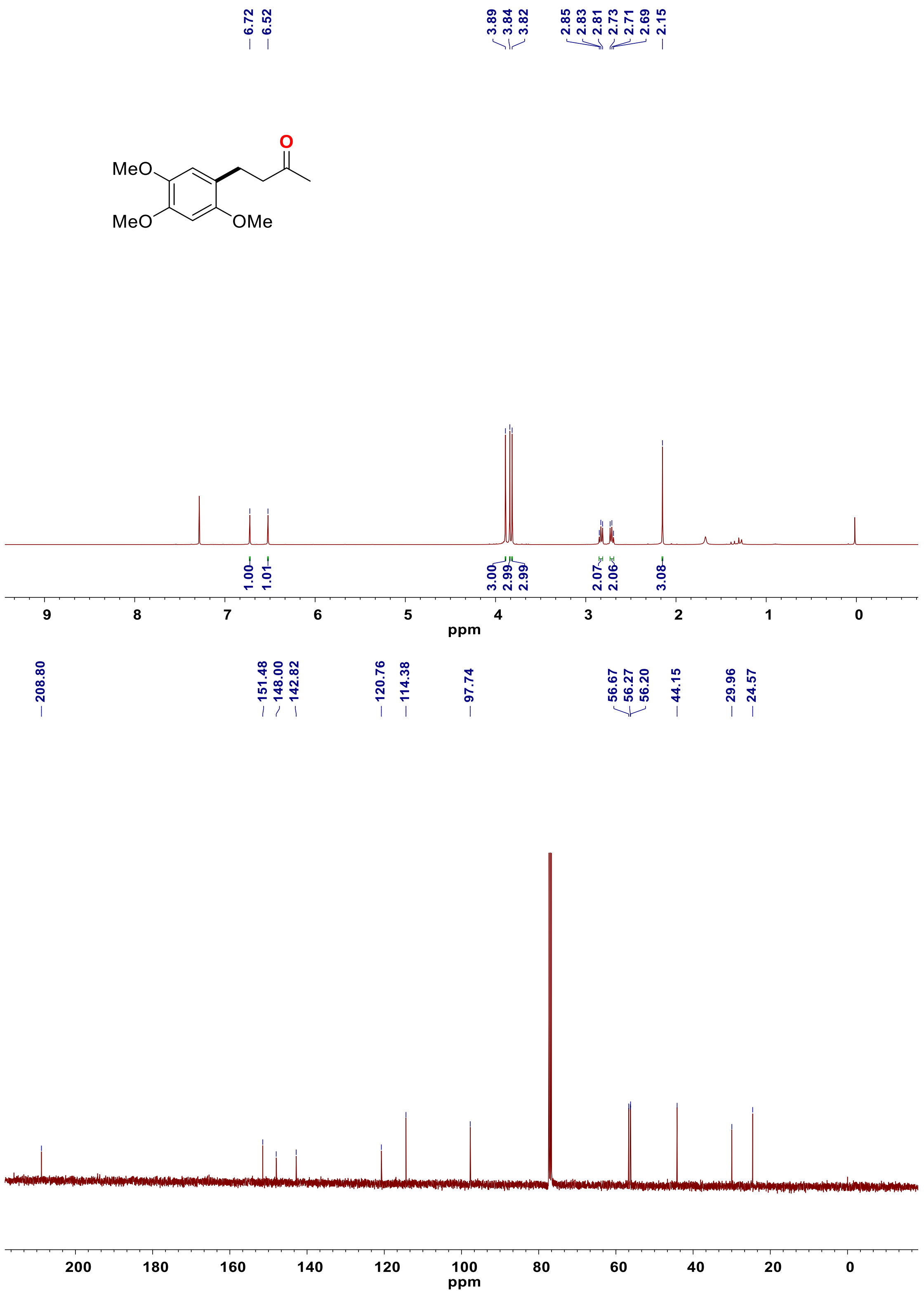

6.72
6.52
3.89
3.84
3.82
2.85
2.83
2.81
2.73
2.71
2.69
2.15
O
MeO
MeO
OMe
1.00
1.01
3.00
2.99
2.99
2.07
2.06
3.08
9 8 7 6 5 4 3 2 1 0
ppm
208.80
151.48
148.00
142.82
120.76
114.38
97.74
56.67
56.27
56.20
44.15
29.96
24.57
200 180 160 140 120 100 80 60 40 20 0
ppm

3b

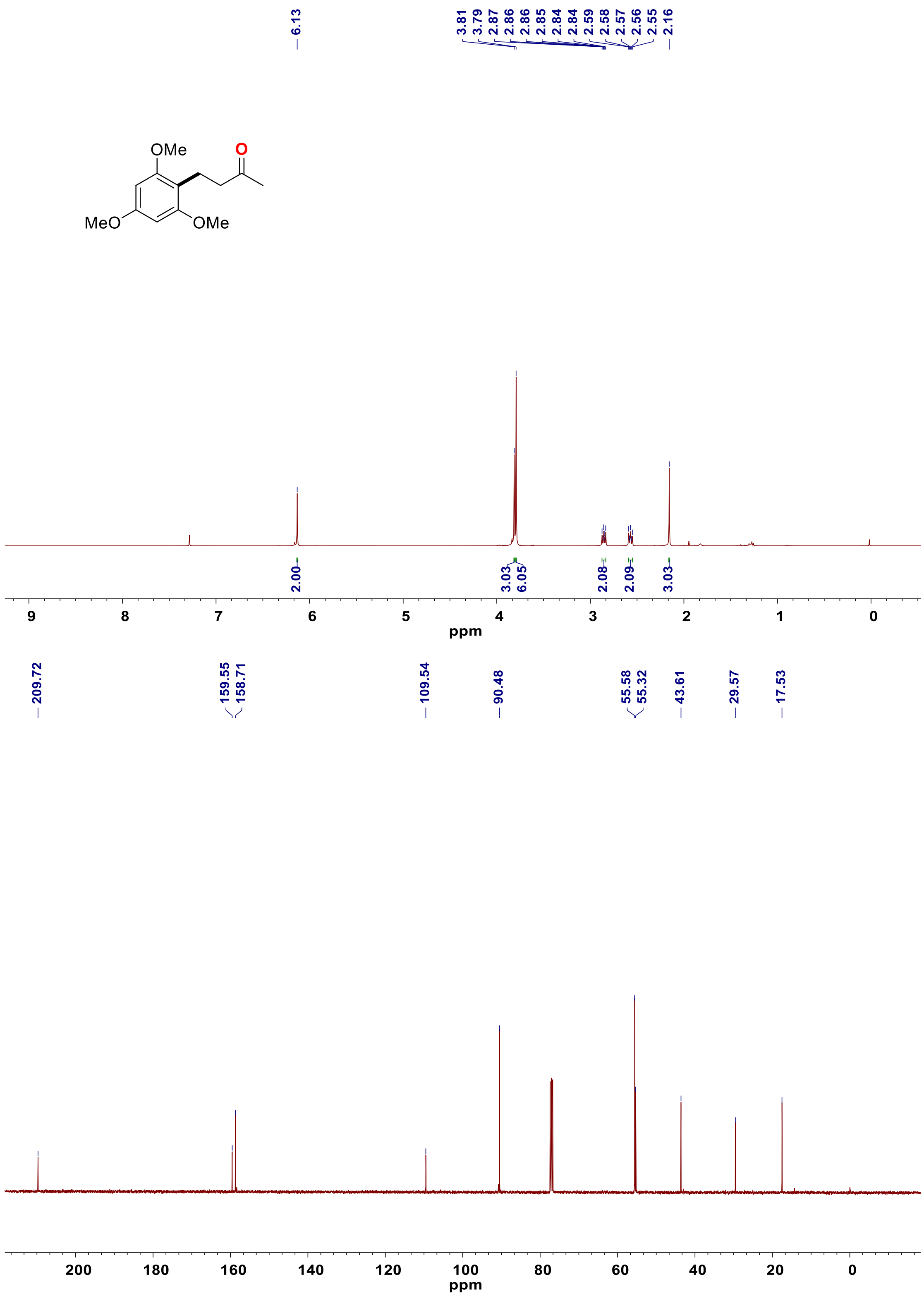

OMe
O
MeO
OMe
6.13
3.81
3.79
2.87
2.86
2.86
2.85
2.84
2.84
2.59
2.58
2.57
2.56
2.55
2.16
2.00
3.03
6.05
2.08
2.09
3.03
ppm
209.72
159.55
158.71
109.54
90.48
55.58
55.32
43.61
29.57
17.53
ppm

3c

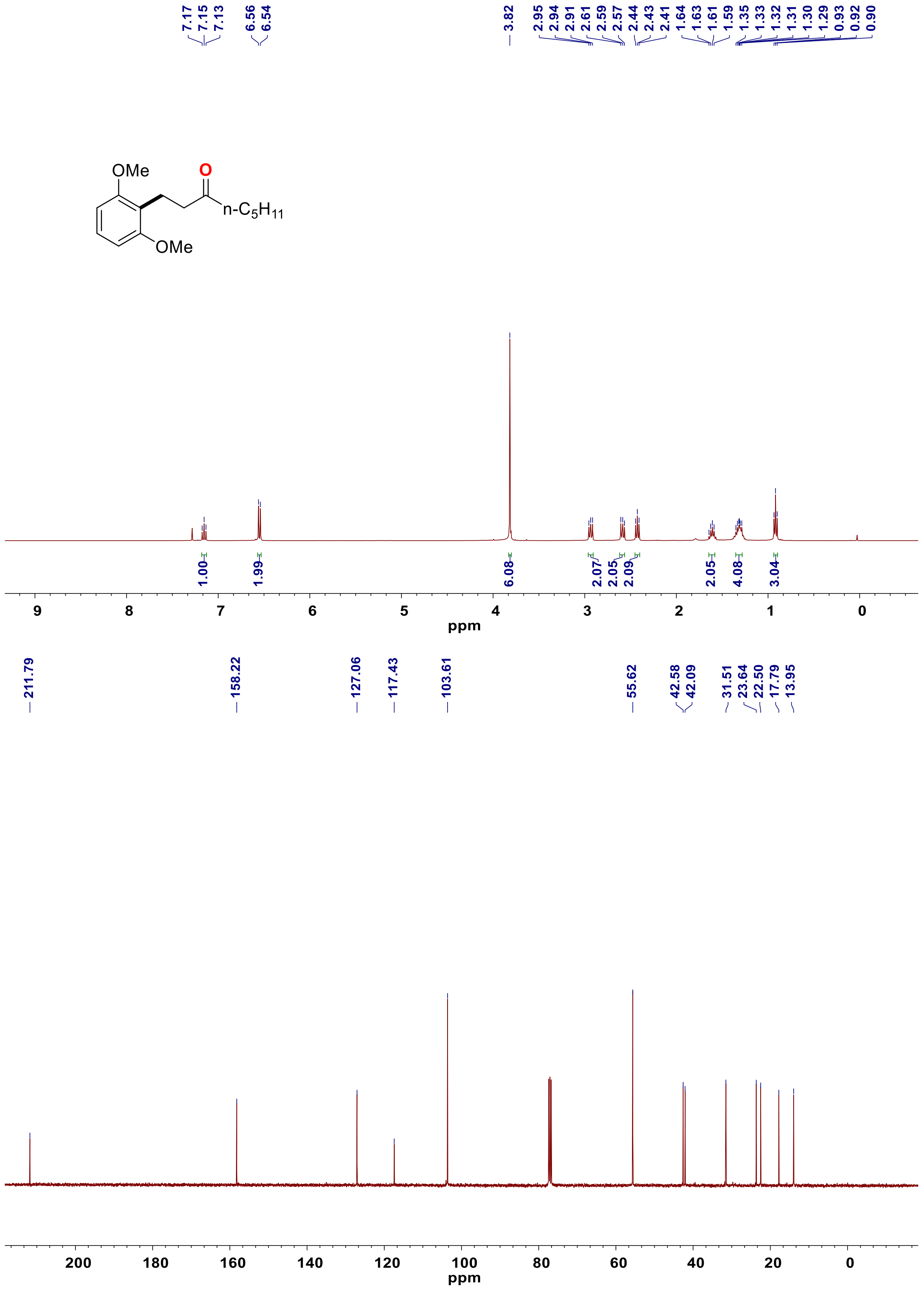

7.17
7.15
7.13
6.56
6.54
3.82
2.95
2.94
2.91
2.61
2.59
2.57
2.44
2.43
2.41
1.64
1.63
1.61
1.59
1.35
1.33
1.32
1.31
1.30
1.29
0.93
0.92
0.90
OMe
O
n-C5H11
OMe
1.00
1.99
6.08
2.07
2.05
2.09
2.05
4.08
3.04
9
8
7
6
5
4
3
2
1
0
ppm
211.79
158.22
127.06
117.43
103.61
55.62
42.58
42.09
31.51
23.64
22.50
17.79
13.95
200
180
160
140
120
100
80
60
40
20
0
ppm

3d

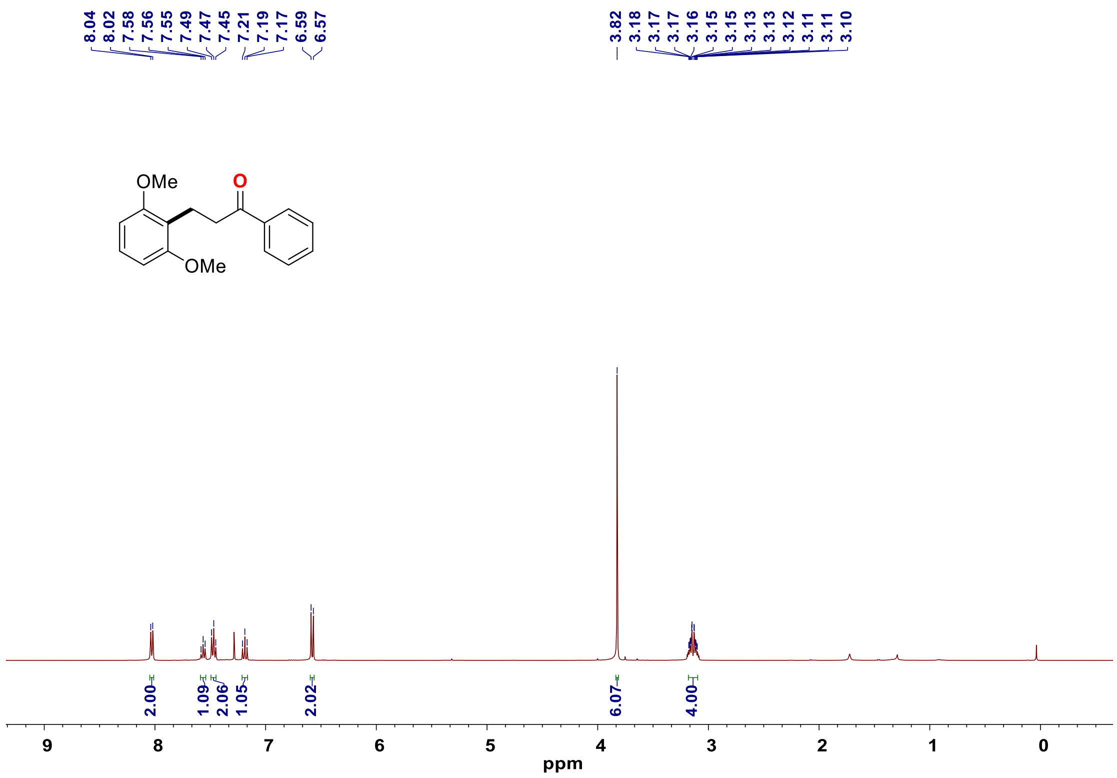
8.04
8.02
7.58
7.56
7.55
7.49
7.47
7.45
7.21
7.19
7.17
6.59
6.57
3.82
3.18
3.17
3.17
3.16
3.15
3.15
3.13
3.13
3.12
3.11
3.11
3.10
OMe
O
OMe
2.00
1.09
2.06
1.05
2.02
6.07
4.00
9
8
7
6
5
4
3
2
1
0
ppm

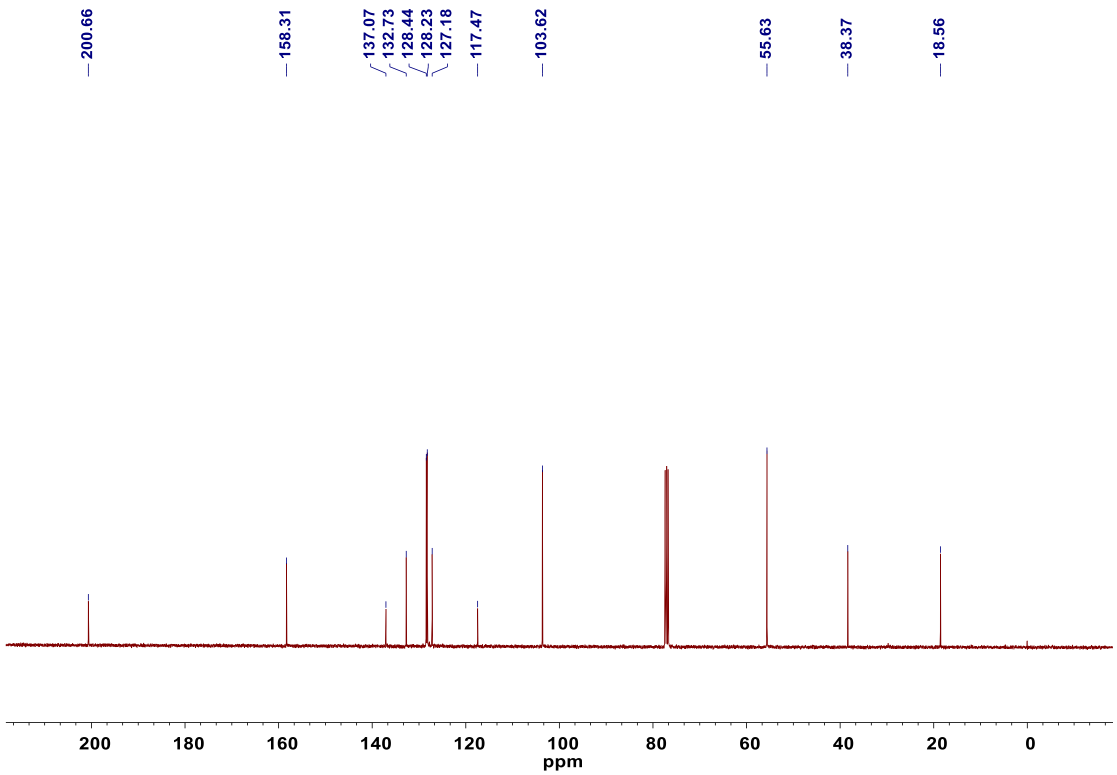
200.66
158.31
137.07
132.73
128.44
128.23
127.18
117.47
103.62
55.63
38.37
18.56
200
180
160
140
120
100
80
60
40
20
0
ppm

3e

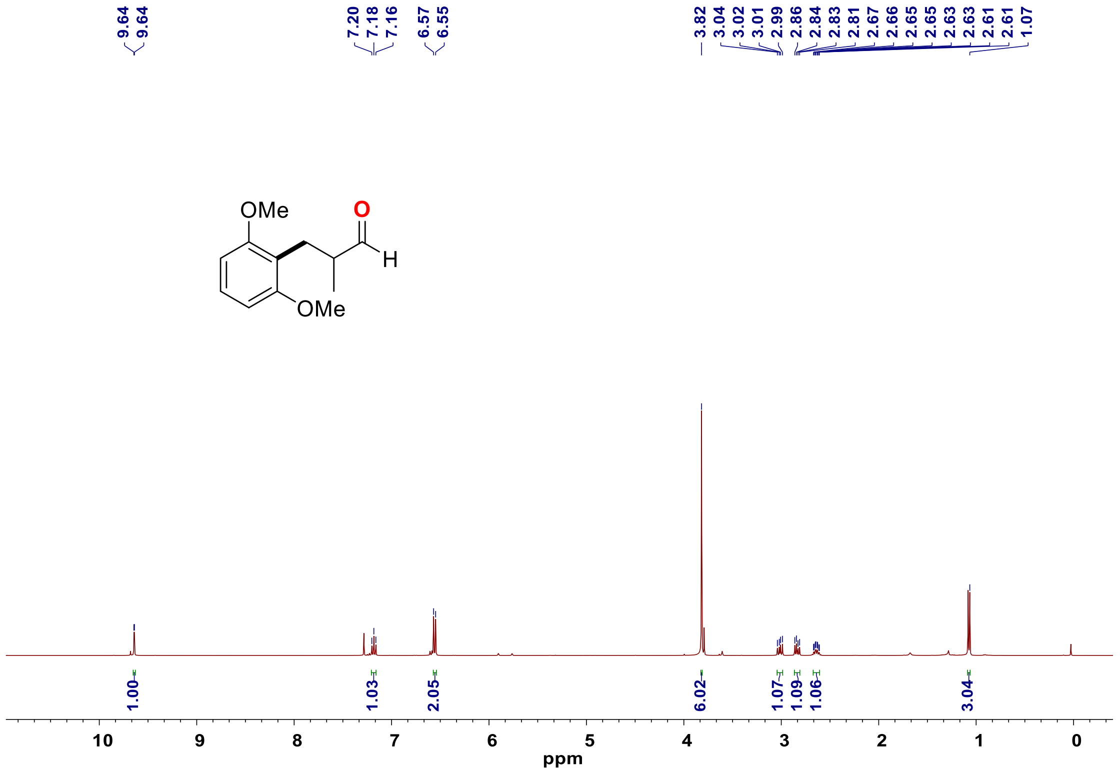
9.64
9.64
7.20
7.18
7.16
6.57
6.55
3.82
3.04
3.02
3.01
2.99
2.86
2.84
2.83
2.81
2.67
2.66
2.65
2.63
2.63
2.61
2.61
1.07
OMe
O
H
OMe
1.00
1.03
2.05
6.02
1.07
1.09
1.06
3.04
10
9
8
7
6
5
4
3
2
1
0
ppm

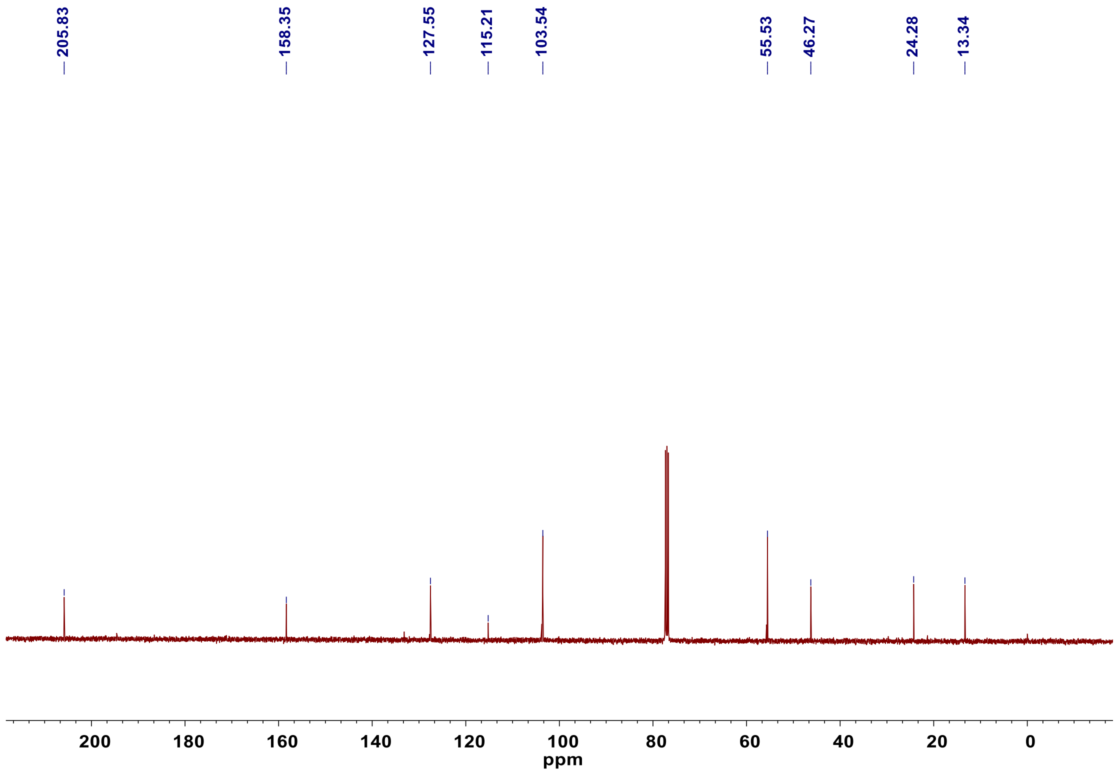
205.83
158.35
127.55
115.21
103.54
55.53
46.27
24.28
13.34
200
180
160
140
120
100
80
60
40
20
0
ppm

3f

^{1}H NMR and ^{13}C NMR spectra of the substrates in Pd-catalyzed acetoxypalladation coupling reaction.

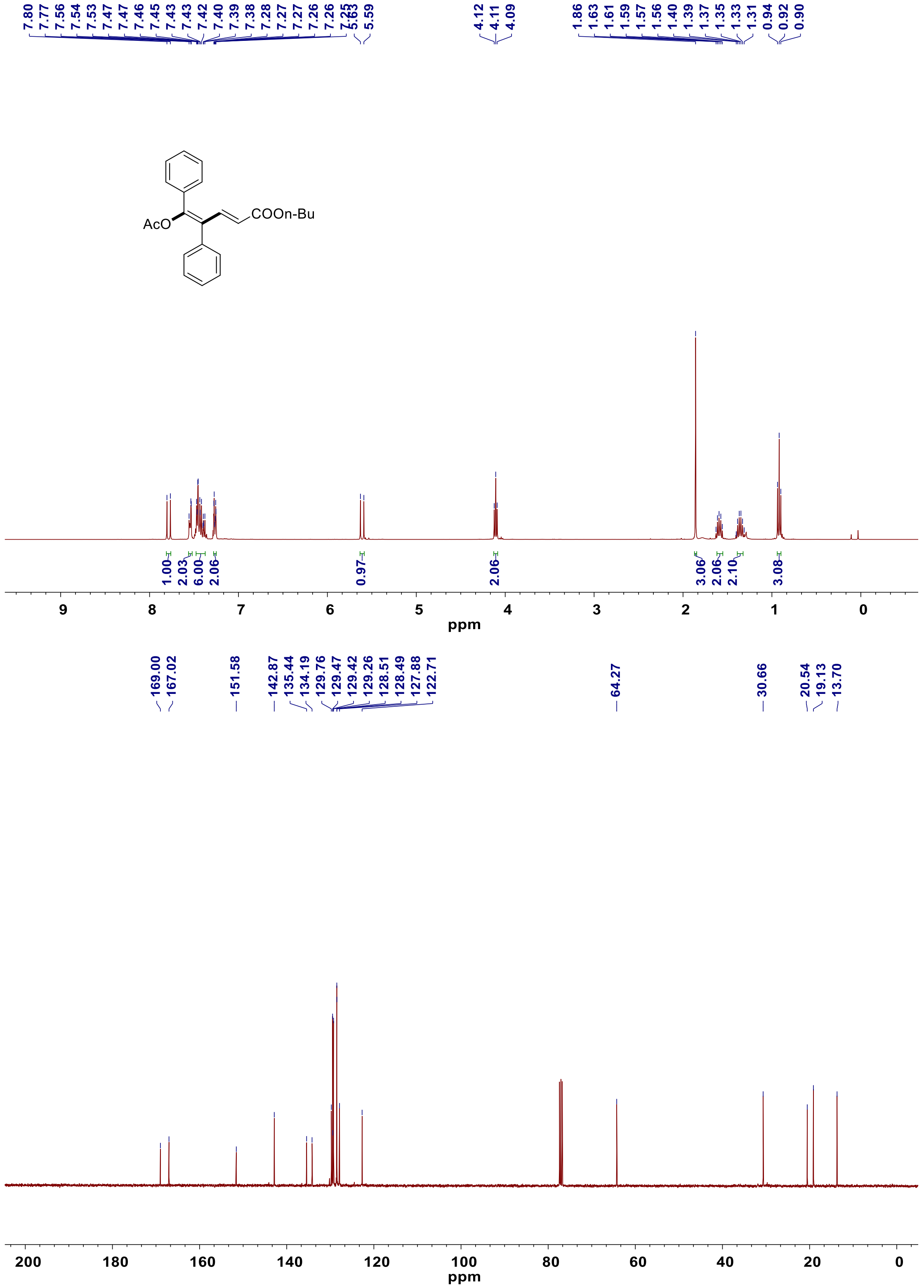

6a

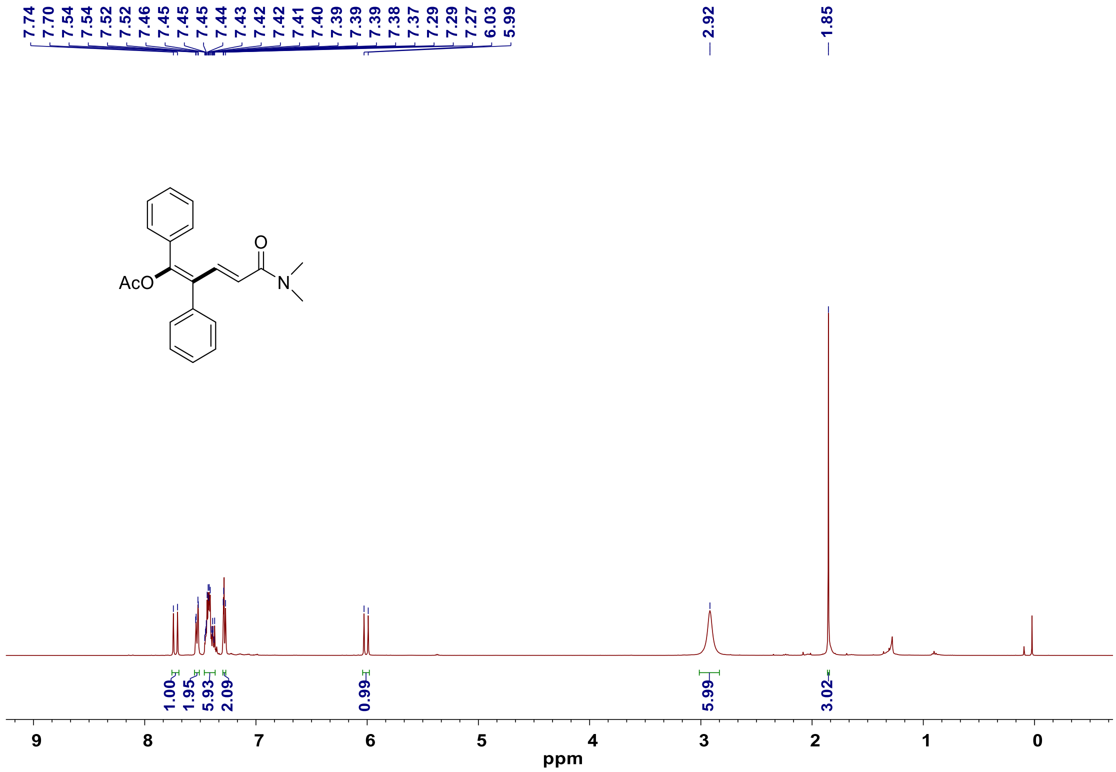
7.74
7.70
7.54
7.54
7.52
7.52
7.46
7.45
7.45
7.45
7.44
7.43
7.42
7.42
7.41
7.40
7.39
7.39
7.39
7.38
7.37
7.29
7.29
7.27
6.03
5.99
2.92
1.85
AcO
O
N
1.00
1.95
5.93
2.09
0.99
5.99
3.02
9
8
7
6
5
4
3
2
1
0
ppm

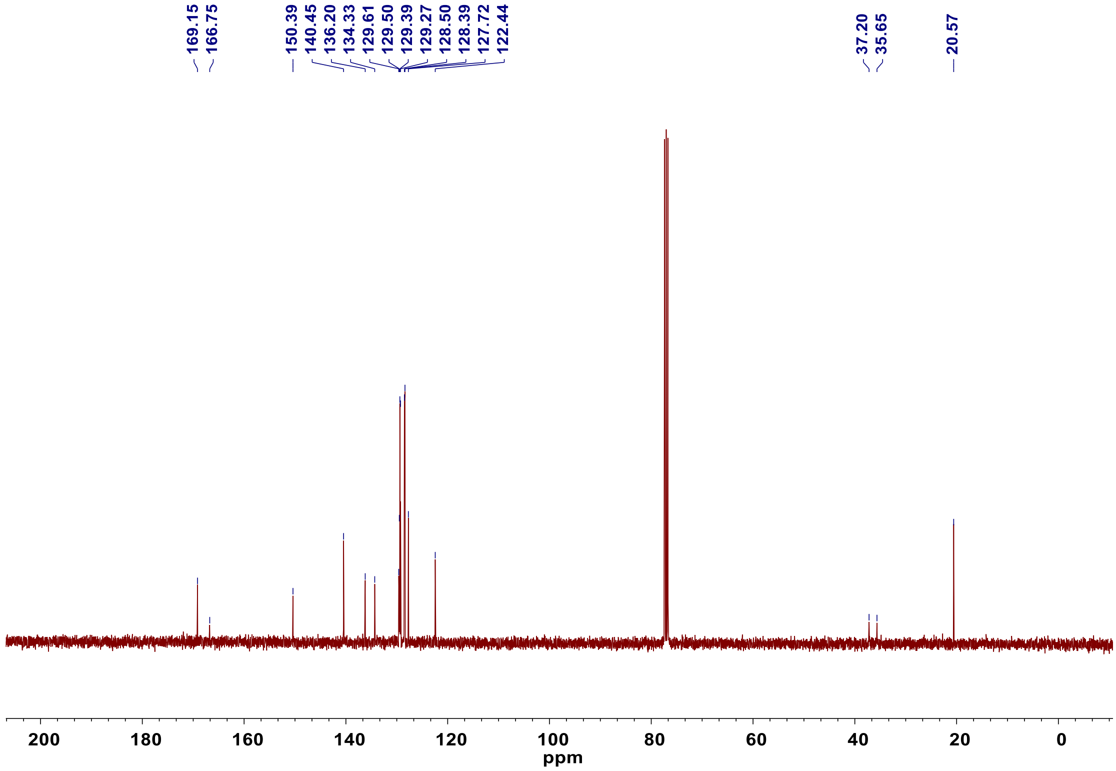
169.15
166.75
150.39
140.45
136.20
134.33
129.61
129.50
129.39
129.27
128.50
128.39
127.72
122.44
37.20
35.65
20.57
200
180
160
140
120
100
80
60
40
20
0
ppm

6b

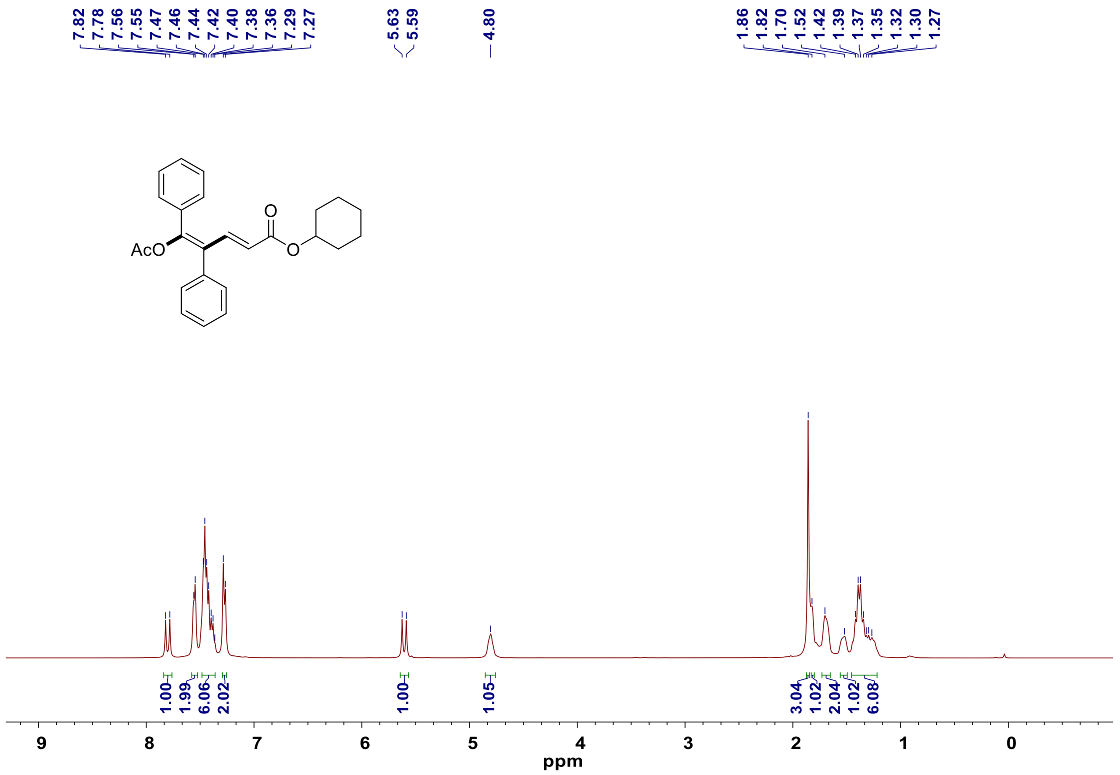

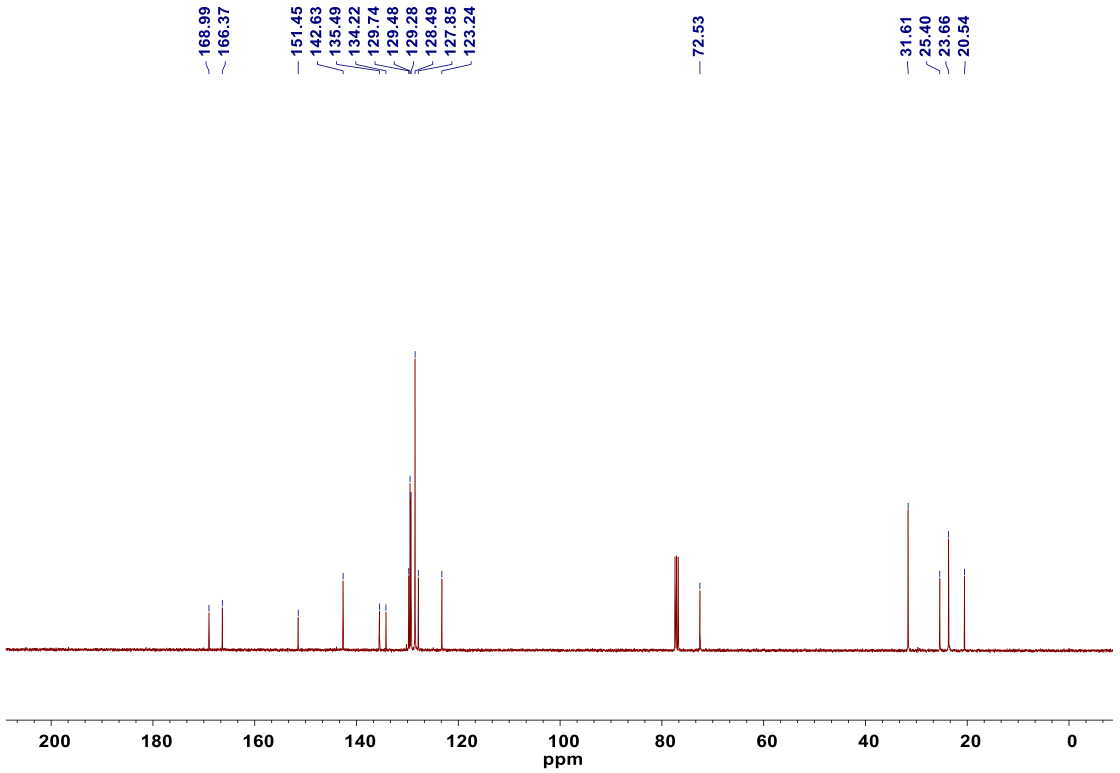

6c

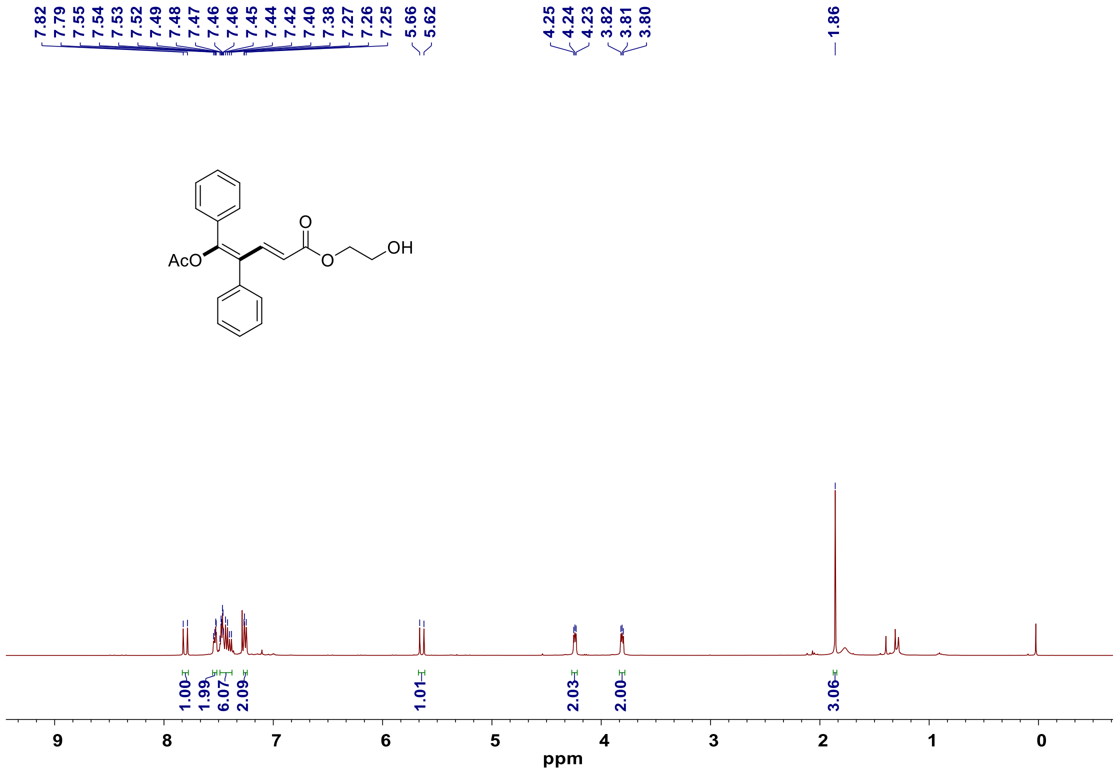
7.82
7.79
7.55
7.54
7.53
7.52
7.49
7.48
7.47
7.46
7.46
7.45
7.44
7.42
7.40
7.38
7.27
7.26
7.25
5.66
5.62
4.25
4.24
4.23
3.82
3.81
3.80
1.86
AcO
O
O
OH
1.00
1.99
6.07
2.09
1.01
2.03
2.00
3.06
9
8
7
6
5
4
3
2
1
0
ppm

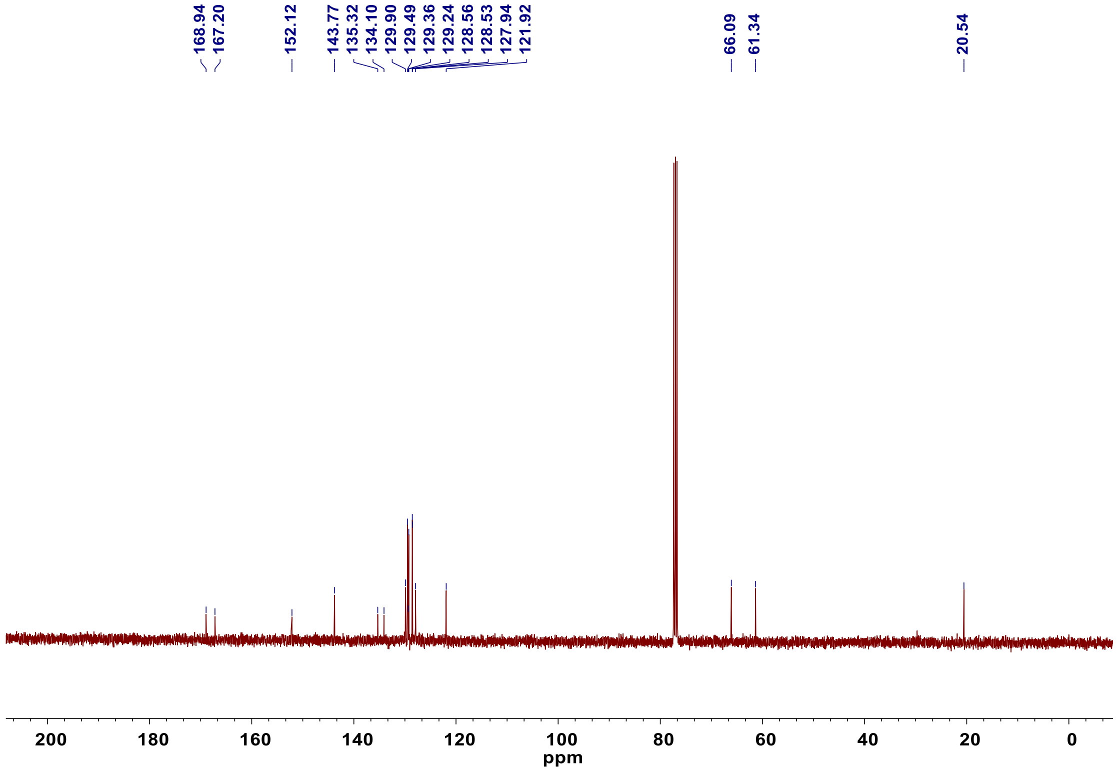
168.94
167.20
152.12
143.77
135.32
134.10
129.90
129.49
129.36
129.24
128.56
128.53
127.94
121.92
66.09
61.34
20.54
200
180
160
140
120
100
80
60
40
20
0
ppm

6d

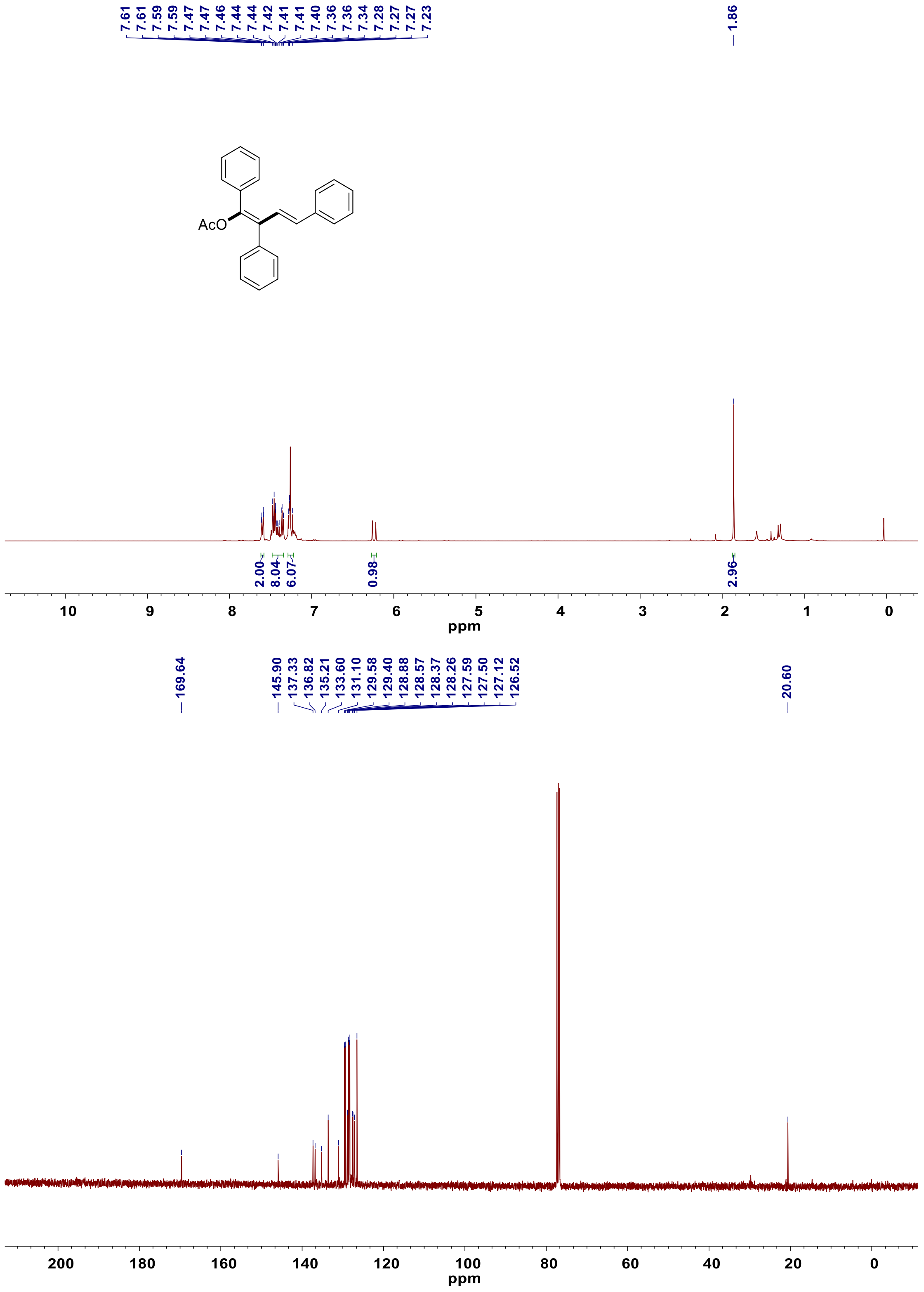
7.61 7.61 7.59 7.59 7.47 7.47 7.46 7.44 7.44 7.42 7.41 7.41 7.40 7.36 7.34 7.28 7.27 7.27 7.23
1.86
AcO
2.00 8.04 6.07 0.98 2.96
10 9 8 7 6 5 4 3 2 1 0
ppm
169.64
145.90 137.33 136.82 135.21 133.60 131.10 129.58 129.40 128.88 128.57 128.37 128.26 127.59 127.50 127.12 126.52
20.60
200 180 160 140 120 100 80 60 40 20 0
ppm

6e

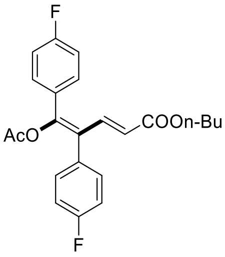
F
AcO
COOn-Bu
F

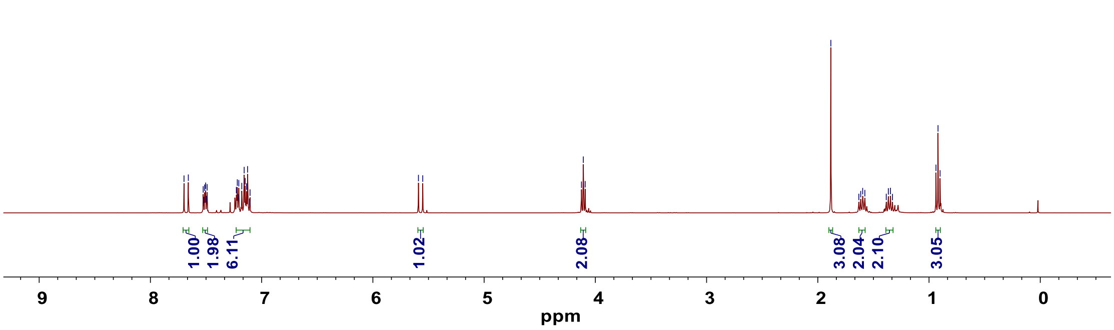
7.70
7.66
7.53
7.52
7.51
7.50
7.50
7.49
7.23
7.22
7.21
7.21
7.18
7.16
7.15
7.14
7.13
7.11
7.10
5.59
5.55
4.12
4.11
4.09
1.88
1.63
1.61
1.60
1.58
1.38
1.37
1.35
1.33
0.94
0.92
0.90
1.00
1.98
6.11
1.02
2.08
3.08
2.04
2.10
3.05
9
8
7
6
5
4
3
2
1
0
ppm

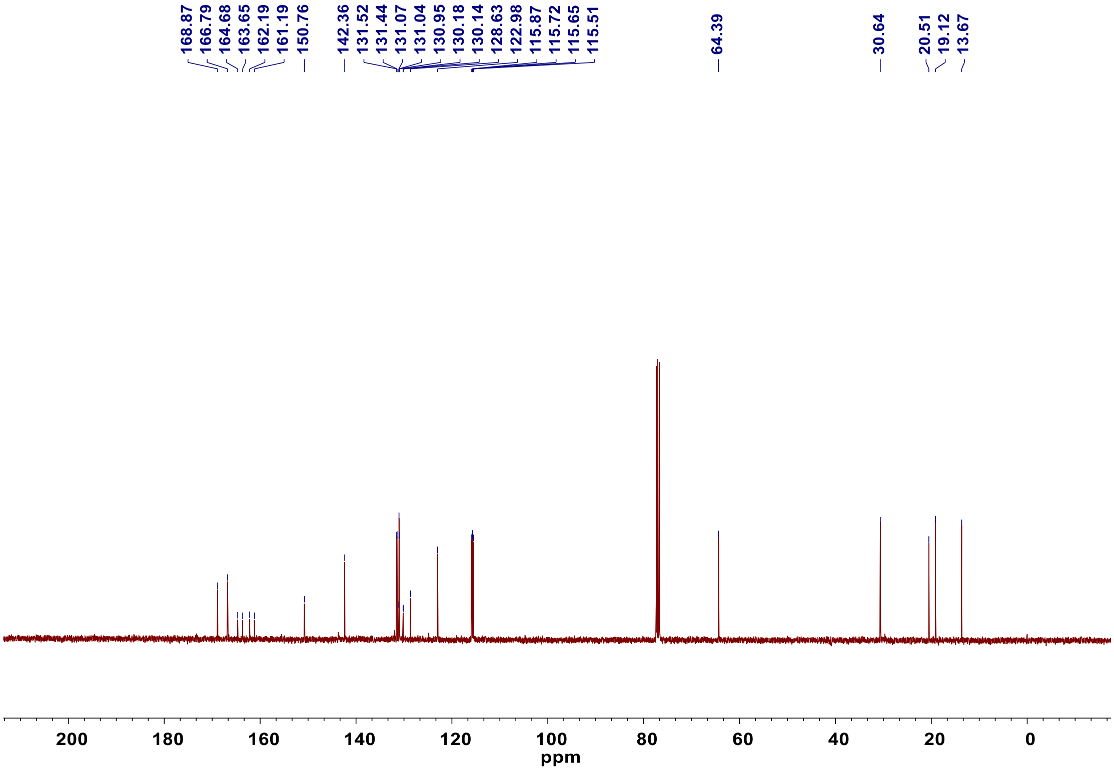
168.87
166.79
164.68
163.65
162.19
161.19
150.76
142.36
131.52
131.44
131.07
131.04
130.95
130.18
130.14
128.63
122.98
115.87
115.72
115.65
115.51
64.39
30.64
20.51
19.12
13.67
200
180
160
140
120
100
80
60
40
20
0
ppm

6f

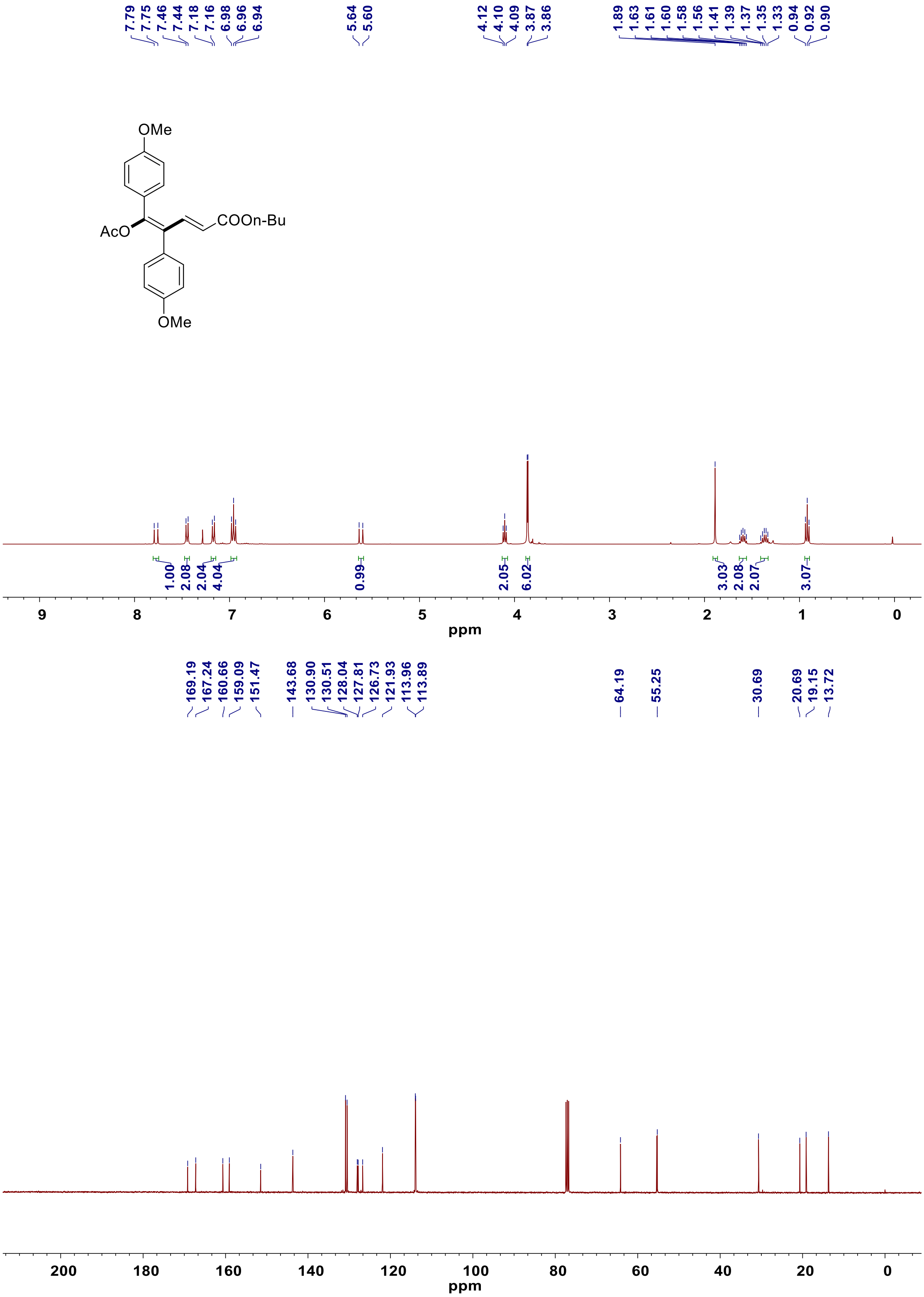
7.79 7.75 7.46 7.44 7.18 7.16 6.98 6.96 6.94
5.64 5.60
4.12 4.10 4.09 3.87 3.86
1.89 1.63 1.61 1.60 1.58 1.56 1.41 1.39 1.37 1.35 1.33 0.94 0.92 0.90
OMe
AcO
COOn-Bu
OMe
1.00 2.08 2.04 4.04
0.99
2.05 6.02
3.03 2.08 2.07
3.07
9 8 7 6 5 4 3 2 1 0
ppm
169.19 167.24 160.66 159.09 151.47
143.68 130.90 130.51 128.04 127.81 126.73 121.93 113.96 113.89
64.19 55.25
30.69
20.69 19.15 13.72
200 180 160 140 120 100 80 60 40 20 0
ppm

6g

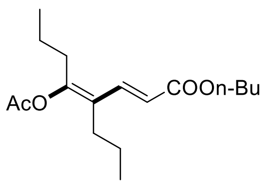
AcO
COOn-Bu

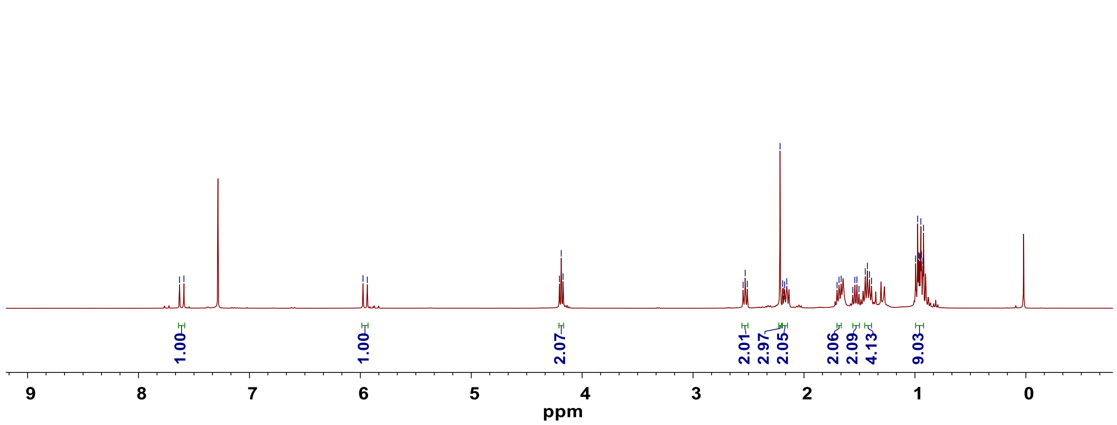
7.63
7.59
5.98
5.94
4.21
4.19
4.17
2.55
2.53
2.51
2.22
2.19
2.18
2.17
2.16
1.68
1.66
1.54
1.52
1.45
1.43
1.41
0.99
0.98
0.97
0.96
0.95
0.94
0.93
1.00
1.00
2.07
2.01
2.97
2.05
2.06
2.09
4.13
9.03
9 8 7 6 5 4 3 2 1 0
ppm

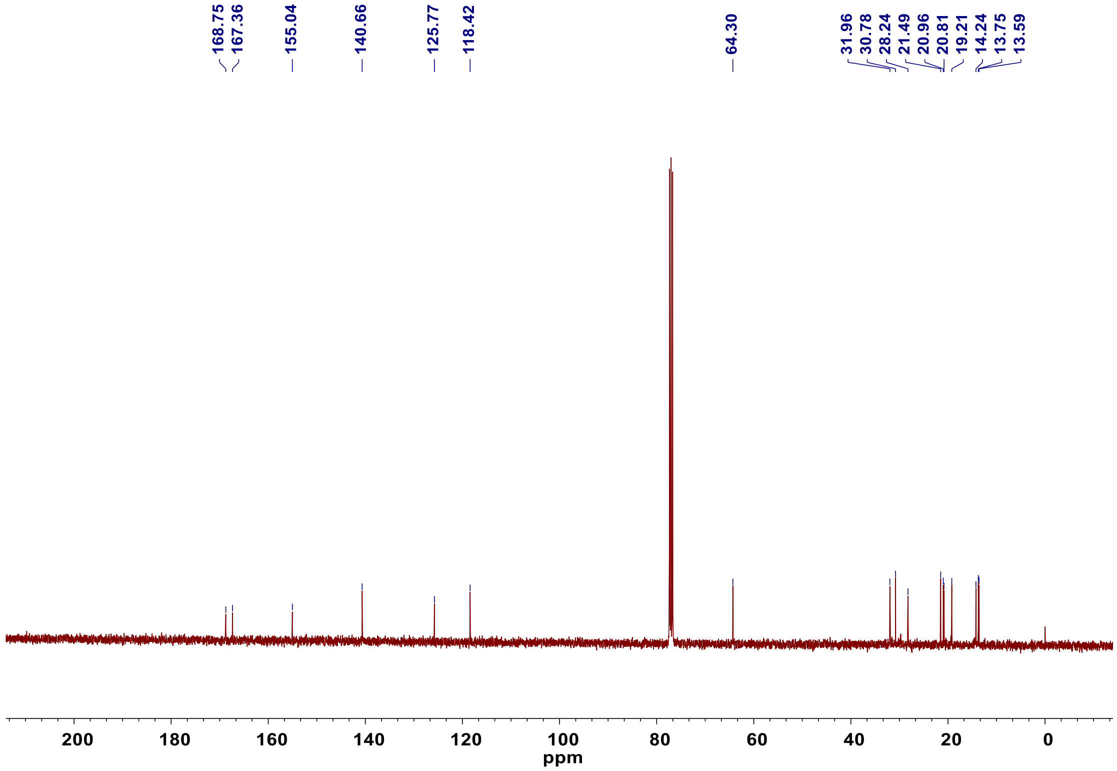
168.75
167.36
155.04
140.66
125.77
118.42
64.30
31.96
30.78
28.24
21.49
20.96
20.81
19.21
14.24
13.75
13.59
200 180 160 140 120 100 80 60 40 20 0
ppm

6h

^{1}H NMR and ^{13}C NMR spectra of the substrates in Pd-catalyzed C−H alkenylation of 2-phenylphenol.

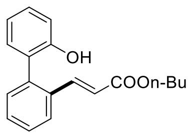

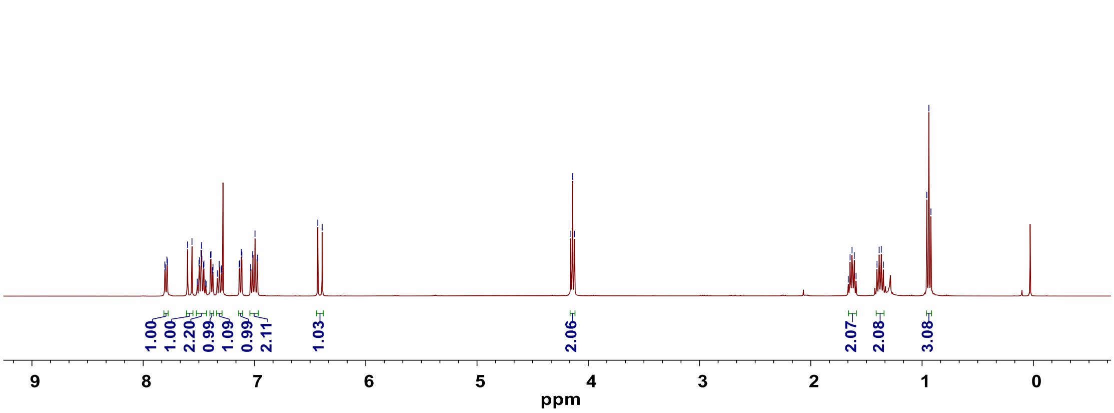

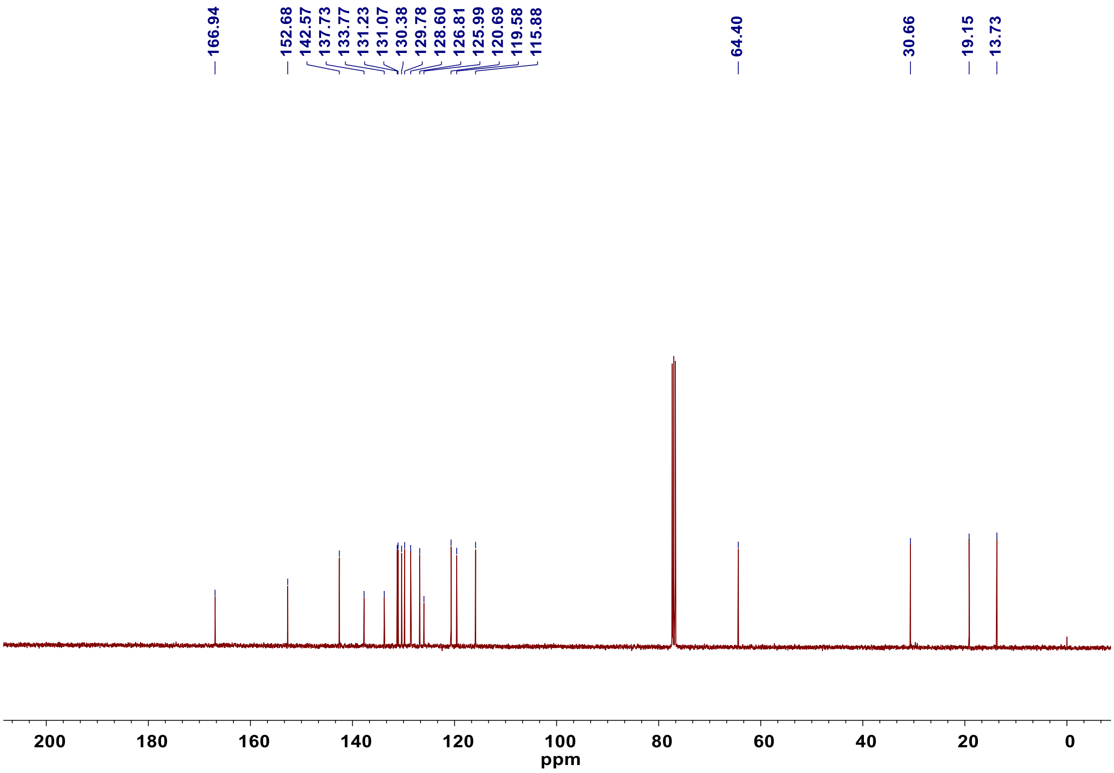

9a

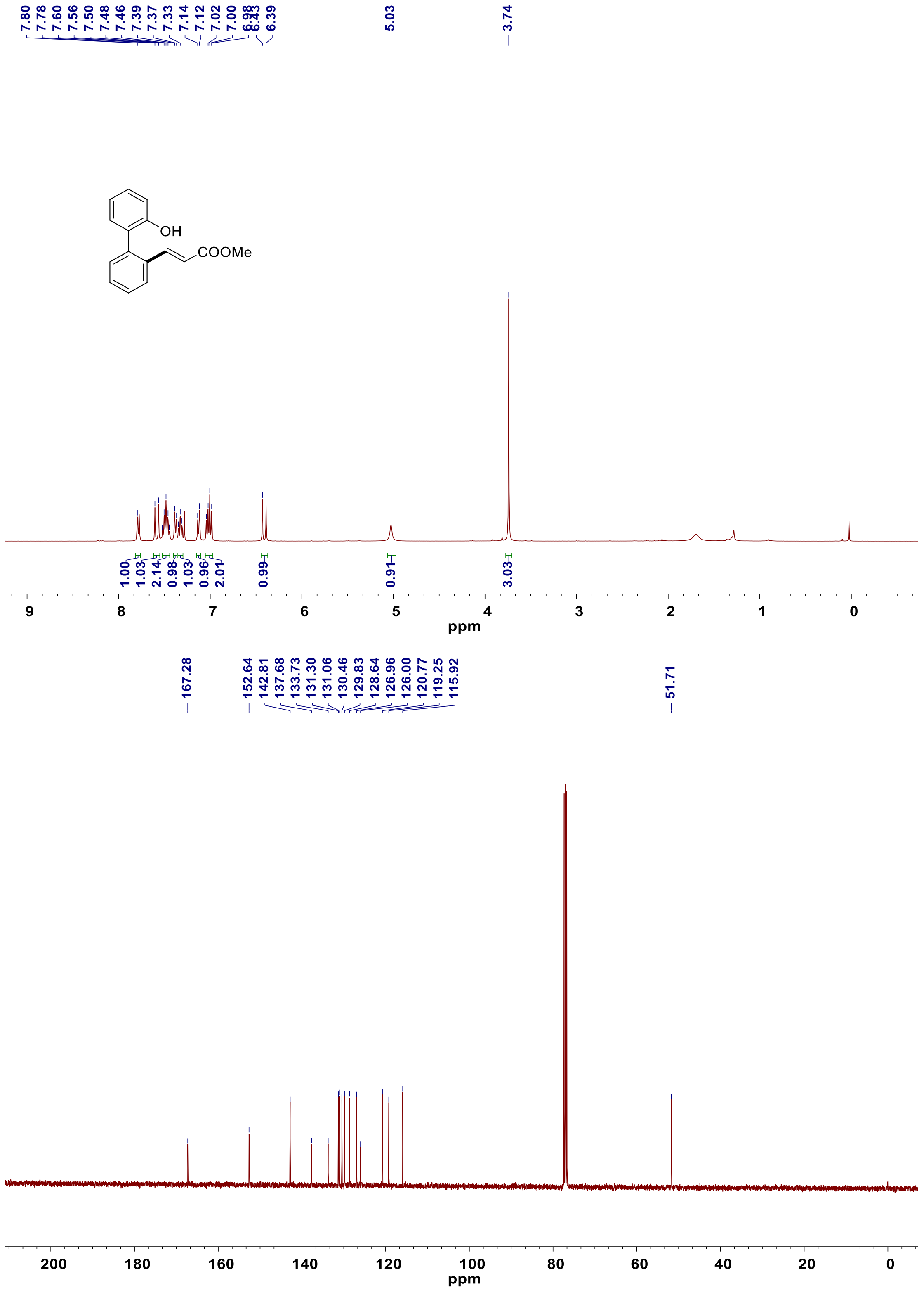

7.80
7.78
7.60
7.56
7.50
7.48
7.46
7.39
7.37
7.33
7.14
7.12
7.02
7.00
6.98
6.43
6.39
5.03
3.74
OH
COOMe
1.00
1.03
2.14
0.98
1.03
0.96
2.01
0.99
0.91
3.03
ppm
167.28
152.64
142.81
137.68
133.73
131.30
131.06
130.46
129.83
128.64
126.96
126.00
120.77
119.25
115.92
51.71
ppm

9b

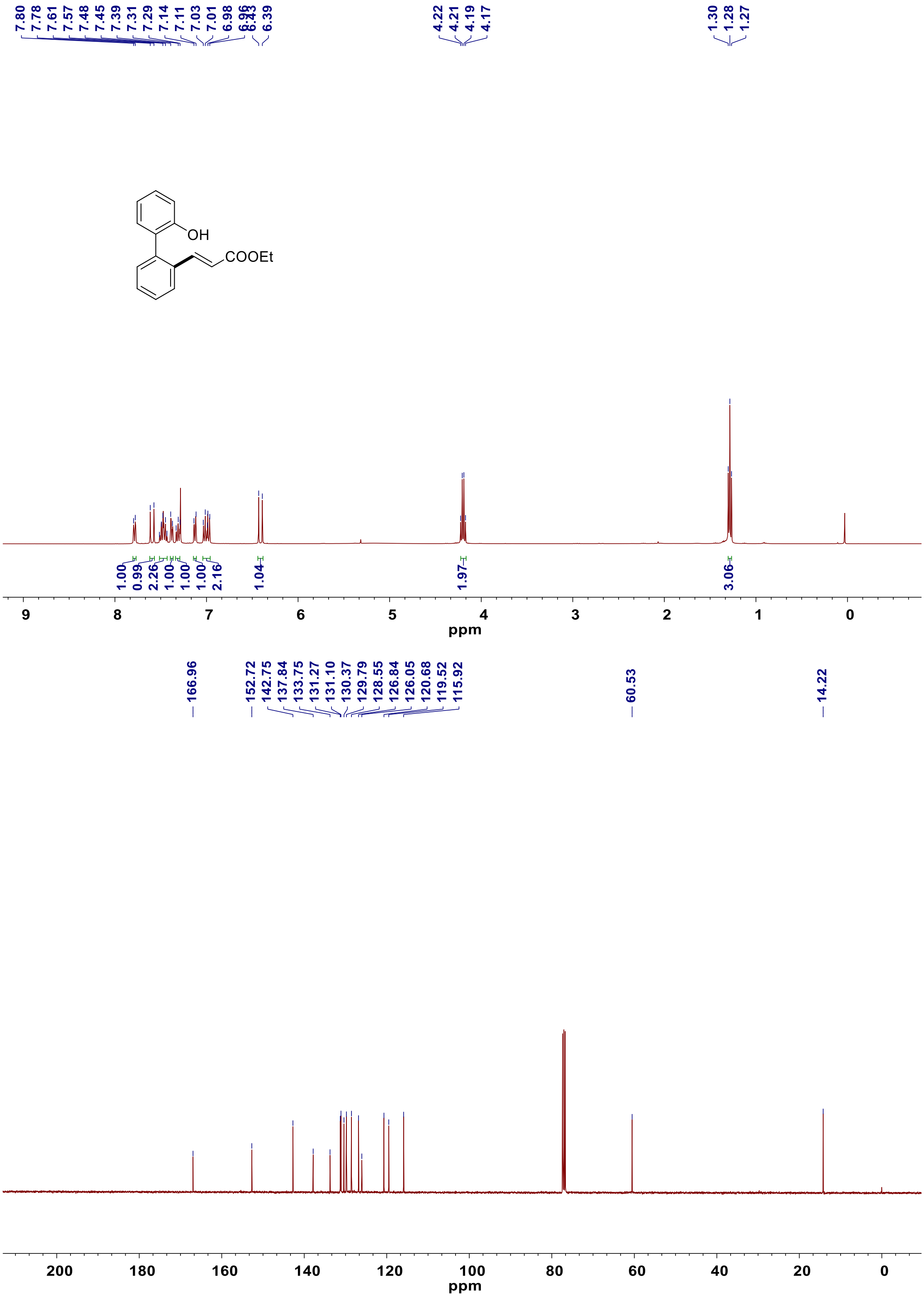
7.80
7.78
7.61
7.57
7.48
7.45
7.39
7.31
7.29
7.14
7.11
7.03
7.01
6.98
6.96
6.43
6.39
4.22
4.21
4.19
4.17
1.30
1.28
1.27
OH
COOEt
1.00
0.99
2.26
1.00
1.00
1.00
2.16
1.04
1.97
3.06
9
8
7
6
5
4
3
2
1
0
ppm
166.96
152.72
142.75
137.84
133.75
131.27
131.10
130.37
129.79
128.55
126.84
126.05
120.68
119.52
115.92
60.53
14.22
200
180
160
140
120
100
80
60
40
20
0
ppm

9c

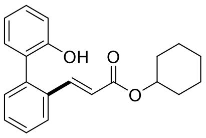
OH
O
O

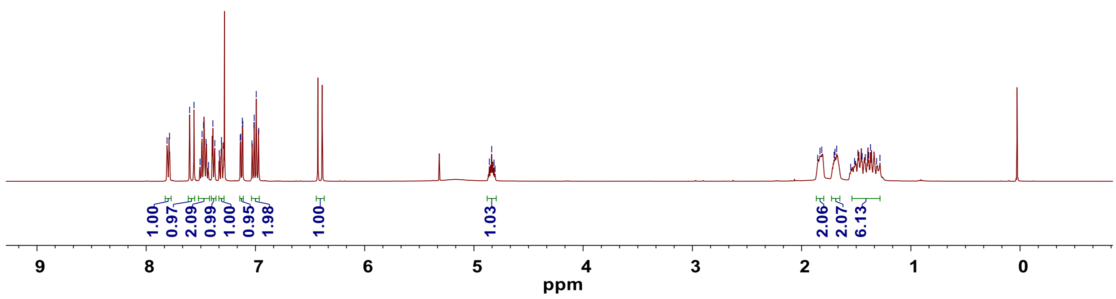
7.81
7.79
7.60
7.56
7.51
7.49
7.47
7.45
7.43
7.39
7.37
7.33
7.31
7.29
7.14
7.13
7.12
7.12
7.03
7.01
7.01
6.99
6.97
4.86
4.84
4.84
4.82
4.81
1.85
1.83
1.82
1.70
1.69
1.68
1.55
1.52
1.48
1.45
1.42
1.39
1.37
1.31
1.28
1.00
0.97
2.09
0.99
1.00
0.95
1.98
1.00
1.03
2.06
2.07
6.13
9
8
7
6
5
4
3
2
1
0
ppm

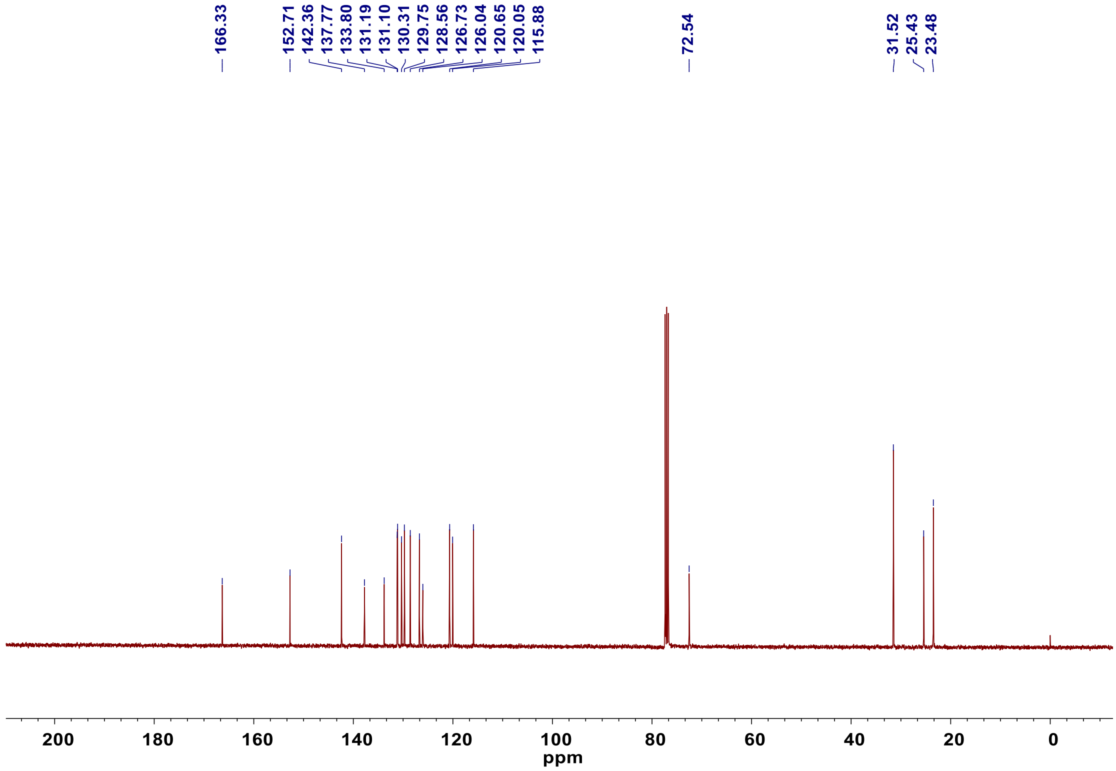
166.33
152.71
142.36
137.77
133.80
131.19
131.10
130.31
129.75
128.56
126.73
126.04
120.65
120.05
115.88
72.54
31.52
25.43
23.48
200
180
160
140
120
100
80
60
40
20
0
ppm

9d

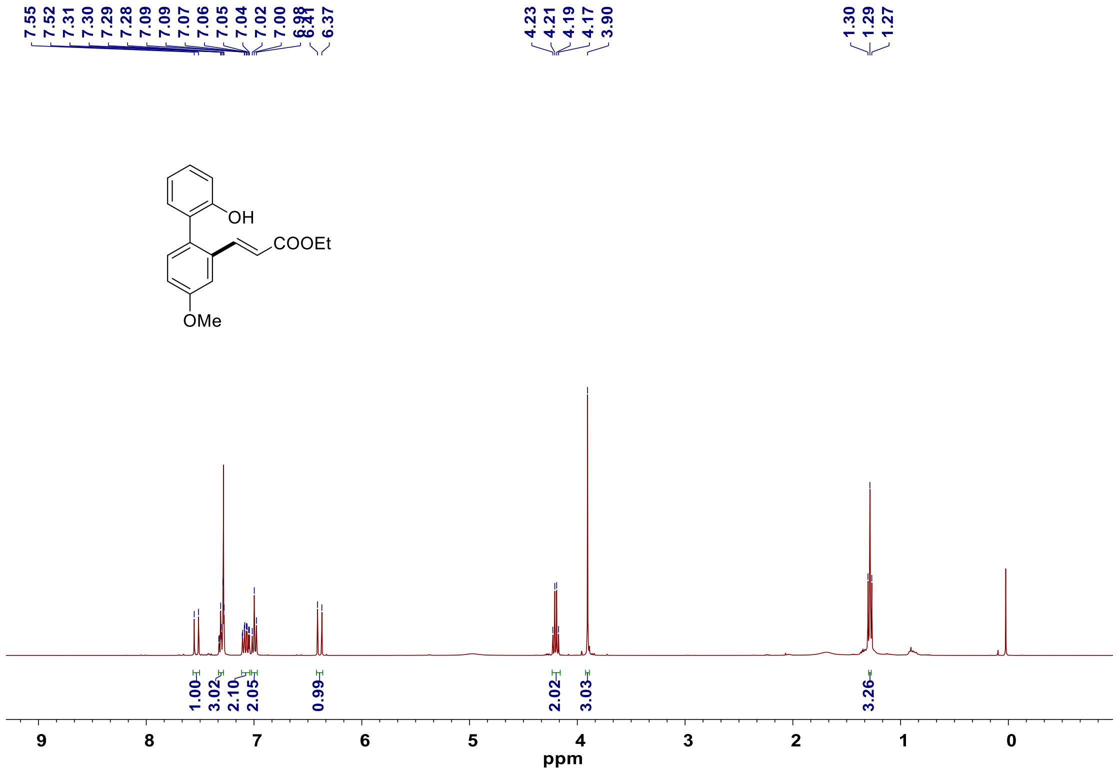

7.55
7.52
7.31
7.30
7.29
7.28
7.09
7.09
7.07
7.06
7.05
7.04
7.02
7.00
6.98
6.41
6.37
4.23
4.21
4.19
4.17
3.90
1.30
1.29
1.27
OH
COOEt
OMe
1.00
3.02
2.10
2.05
0.99
2.02
3.03
3.26
9
8
7
6
5
4
3
2
1
0
ppm

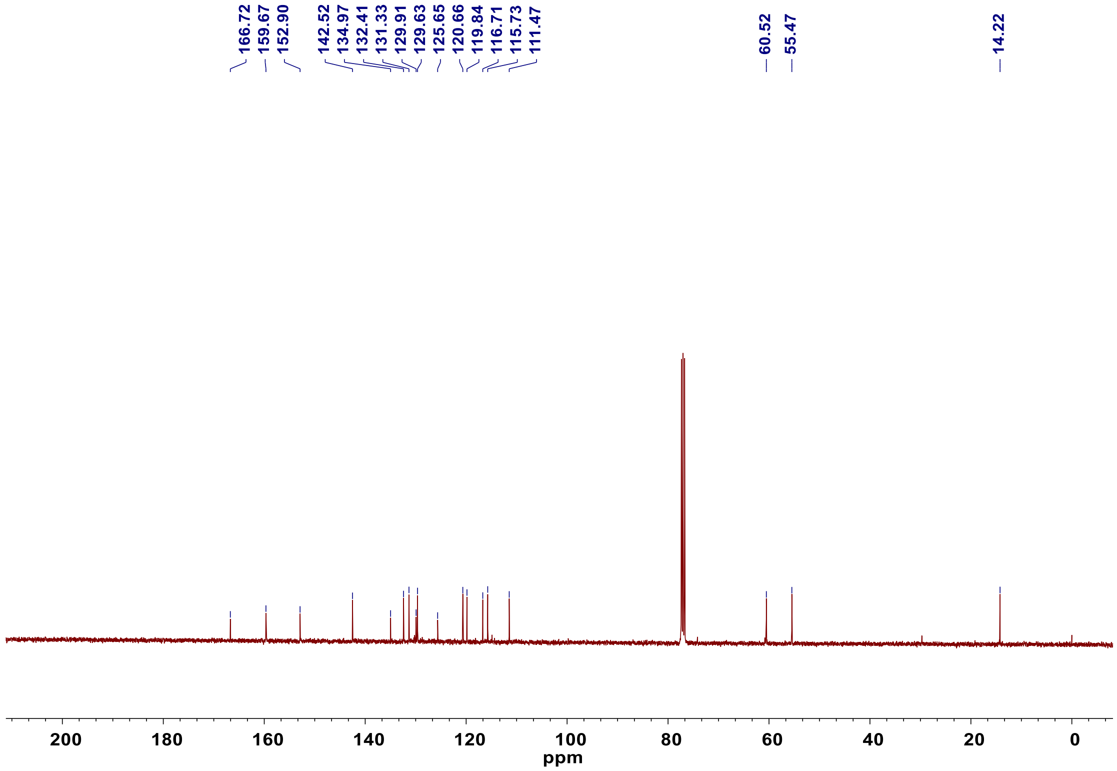

166.72
159.67
152.90
142.52
134.97
132.41
131.33
129.91
129.63
125.65
120.66
119.84
116.71
115.73
111.47
60.52
55.47
14.22
200
180
160
140
120
100
80
60
40
20
0
ppm

9e

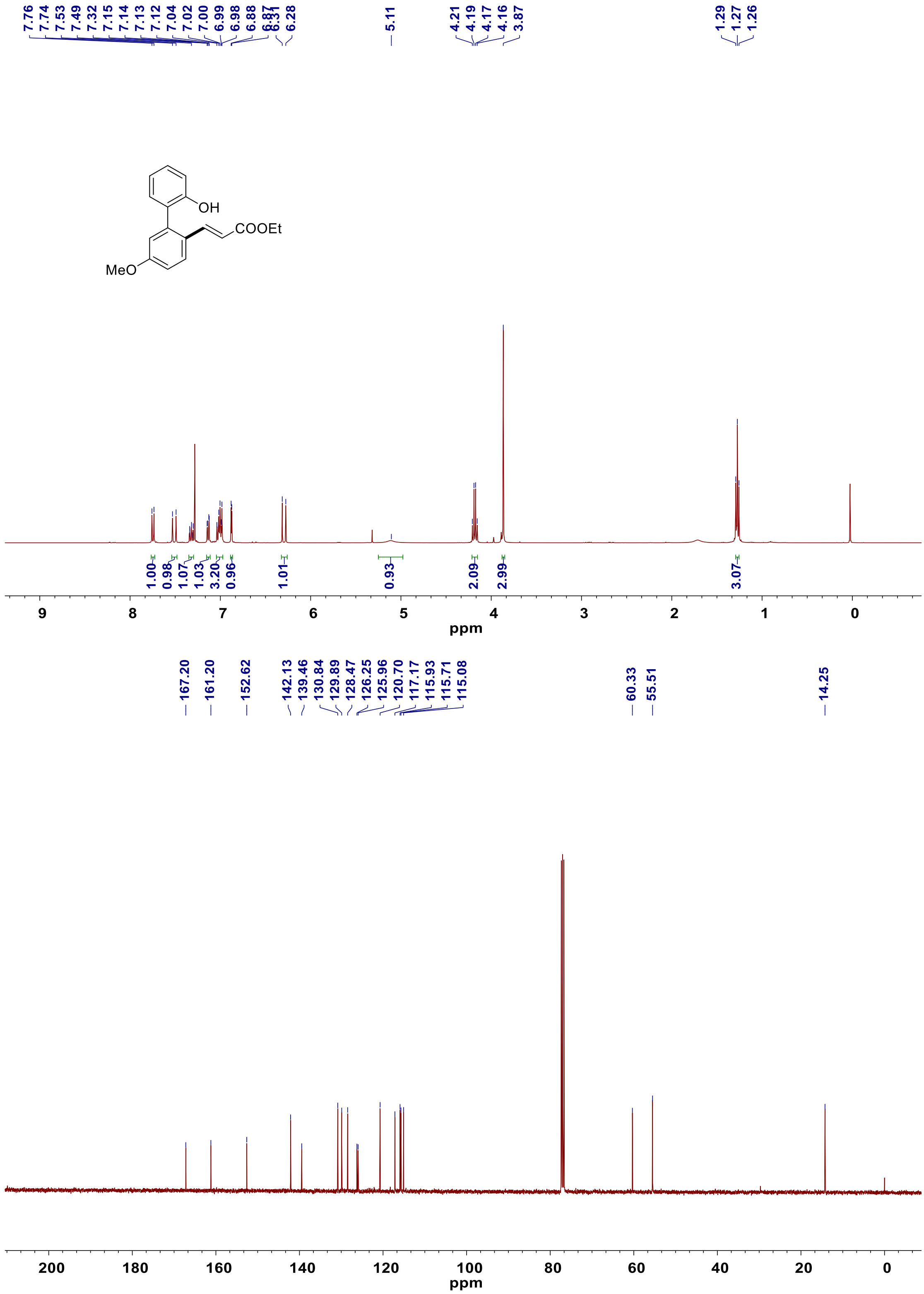
7.76 7.74 7.53 7.49 7.32 7.15 7.14 7.13 7.12 7.04 7.02 7.00 6.99 6.98 6.88 6.87 6.31 6.28
5.11
4.21 4.19 4.17 4.16 3.87
1.29 1.27 1.26
OH
COOEt
MeO
1.00 0.98 1.07 1.03 3.20 0.96 1.01 0.93 2.09 2.99 3.07
9 8 7 6 5 4 3 2 1 0
ppm
167.20 161.20 152.62 142.13 139.46 130.84 129.89 128.47 126.25 125.96 120.70 117.17 115.93 115.71 115.08
60.33 55.51 14.25
200 180 160 140 120 100 80 60 40 20 0
ppm

9f

Supplementary References

[1] Hedley, G. J.; Ruseckas, A.; Samuel, I. D. W. Vibrational Energy Flow Controls Internal Conversion in a Transition Metal Complex. *J. Phys. Chem. A* **114**, 8961-8968 (2010).

[2] Spaenig, F.; Olivier, J. H.; Prusakova, V.; Retailleau, P.; Ziessel, R.; Castellano, F. N. Excited-State Properties of Heteroleptic Iridium(III) Complexes Bearing Aromatic Hydrocarbons with Extended Cores. *Inorg. Chem.* **50**, 10859-10871 (2011).

[3] Hofbeck, T.; Yersin, H. The Triplet State of fac-Ir(ppy)$_3$. *Inorg. Chem.* **49**, 9290-9299 (2010).

[4] Chen, L. Y.; Chen, H. R.; Luque, R.; Li, Y. W. Metal-organic Framework Encapsulated Pd Nanoparticles: Towards Advanced Heterogeneous Catalysts. *Chem. Sci.* **5**, 3708-3714 (2014).

[5] Yarulin, A.; Yuranov, I.; Lizana, F. C.; Abdulkin, P., Minsker, L. K., Size-Effect of Pd-(Poly(N-vinyl-2-pyrrolidone)) Nanocatalysts on Selective Hydrogenation of Alkynols with Different Alkyl Chains. *J. Phys. Chem. C* **117**, 13424-13434 (2013).
